# Initiation of meiosis from human iPSCs under defined conditions through identification of regulatory factors

**DOI:** 10.1126/sciadv.adu0384

**Published:** 2025-08-15

**Authors:** Merrick Pierson Smela, Jessica Adams, Carl Ma, Laura Breimann, Ursula Widocki, Bogdan Dobre, Toshihiro Shioda, George M. Church

**Affiliations:** ^1^ Wyss Institute, Harvard University, Boston, MA 02215, USA.; ^2^ Ovelle Bio, Boston, MA 02115, USA.; ^3^ Department of Genetics, Harvard Medical School, Boston, MA 02115, USA.; ^4^ Broad Institute of MIT and Harvard, Cambridge, MA 02138, USA.; ^5^ Krantz Family Center for Cancer Research, Massachusetts General Hospital, Charlestown, MA 02129, USA.

## Abstract

Meiotic failure is a major cause of infertility, but the lack of an in vitro model of human meiosis is a barrier to understanding its mechanism. Here, we establish a method to initiate meiosis directly from male or female human-induced pluripotent stem cells (iPSCs). DNMT1 inhibition, retinoid signaling activation, and overexpression of regulatory factors (antiapoptotic BCL2 and promeiotic HOXB5, BOLL, or MEIOC) rapidly activates meiosis over a 15-day protocol. Our protocol bypasses the primordial germ cell stage and directly generates cells expressing genes similar to meiotic oogonia, including oogonia markers, all synaptonemal complex components, and meiotic recombination machinery. DNMT1 inhibition rapidly erases DNA methylation, including at imprinting control regions and promoters of meiotic genes. Microscopy shows key aspects of meiosis, including chromosome axis formation and synapsis in live human cells. Our model of human meiosis provides opportunities for studying this critical reproductive process under chemically defined conditions in vitro.

## Teaser

Overexpression of regulatory factors and treatment with small molecules drives human stem cells to directly initiate meiosis.

## INTRODUCTION

All sexually reproducing species rely on meiosis to produce haploid gametes from diploid germ cells. To date, the most detailed studies of meiosis have taken place in nonhuman organisms, due to the lack of a reliable in vitro model of human meiosis, as well as technical and ethical barriers to obtaining meiotic cells from humans. Therefore, a method of inducing meiosis in cultured human cells could greatly advance the study of this crucial reproductive process and could also lead to transformative therapies for people with infertility.

In vivo, meiosis occurs in female oogonia and male spermatocytes, both of which develop from primordial germ cells (PGCs) (*1*). Research on animals such as mice has revealed regulatory mechanisms of mammalian germline development and meiosis, including requirements for erasing DNA methylation (*1*–*3*) and signaling from gonadal somatic cells including retinoic acid and bone morphogenetic protein (*4*). Recent studies have demonstrated the initiation of meiosis in mouse cells in vitro (*4*–*10*), even producing viable offspring from the resulting gametes (*5*, *6*). These methods for mouse in vitro gametogenesis rely on differentiating pluripotent stem cells to PGC-like cells, followed by coculture with gonadal somatic cells to achieve epigenetic resetting, and induction of meiosis by retinoic acid treatment.

For human cells, the most recent state-of-the-art method for recreating germline development in vitro involves differentiating human-induced pluripotent stem cells (hiPSCs) to PGC-like cells, followed by culturing these cells over 120 days. During this time, they erase their DNA methylation and develop to an oogonia-like stage (*11*). This method is an important advance for modeling human oogonia development, and some cells even express low levels of early meiotic genes, but actual meiosis does not occur. Earlier studies also investigated the possibility of meiosis from hiPSCs in vitro (*12*–*17*). These studies activated expression of some meiotic markers including SYCP3 and γH2AX, but the results were inconsistent with the expected localization of these proteins during the different stages of meiosis (*18*), they did not demonstrate transcriptome-wide similarity to meiotic germ cells in vivo, and a contemporaneous review concluded that they did not reach the “gold standard” of inducing meiosis (*19*). Although previous studies correctly identified BOLL as a promeiotic factor (*14*, *17*), they lacked an efficient means of erasing genome-wide DNA methylation, which is a known prerequisite for mammalian meiosis (*1*, *2*). Some studies examined methylation at certain imprinted loci but found only a moderate decrease (*13*, *14*), or even an increase (*15*), from the starting hiPSCs.

Here, we present a direct method for generating meiotic cells from hiPSCs without relying on an intermediate PGC-like stage. By screening conditions for activating the expression of meiotic genes, we show that DNMT1 inhibition, retinoic acid receptor activation, and overexpression of antiapoptosis (BCL2) and promeiosis (MEIOC, BOLL, or HOXB5) factors can rapidly initiate meiosis in male and female hiPSCs. By single-cell RNA sequencing (scRNA-seq), ~16 to 22% of cells across three cell lines tested were annotated as meiotic oogonia, as defined by comparison to a human fetal gonad reference atlas (*20*). Our method produces cells with gene expression similar to meiotic oogonia in vivo and with low levels of DNA methylation. Microscopy of whole cells and chromosome spreads shows leptotene and zygotene stages of meiosis. We also occasionally observe cells with pachytene-like features, including sex body formation in male cells and synaptonemal complex (SC) central element formation, although defects in synapsis prevent progression to later stages of meiosis. Last, we demonstrate imaging of meiotic chromosome synapsis in live human cells. Overall, our method will be a useful tool for researchers studying human meiosis and, with further optimization, may allow the production of human gametes in vitro.

## RESULTS

### Barcode enrichment screening of candidate meiosis-promoting factors

**Fig. 1.**
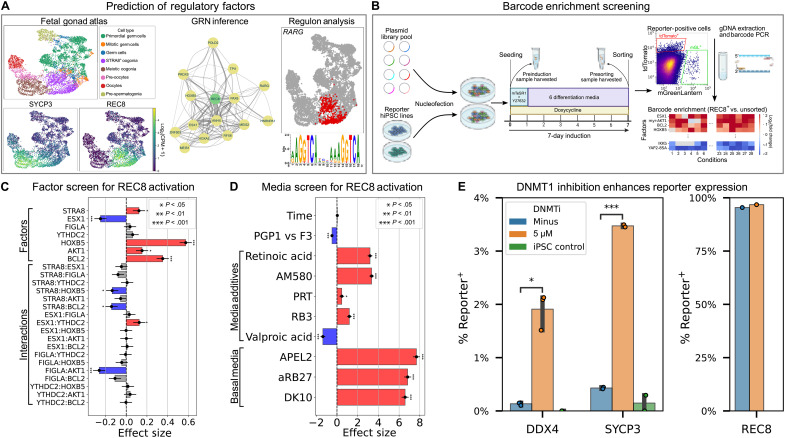
Screening of regulatory factors for reporter activation. (**A**) Prediction of regulatory factors based on fetal gonad scRNA-seq data. *REC8* and *SYCP3* were chosen as reporters due to high expression [counts per million (CPM)] in pre-meiotic and meiotic cells, respectively. (**B**) Barcode enrichment screening in reporter iPSCs (see Materials and Methods). (**C**) Fractional factorial screen (32 combinations, each tested in two cell lines) of seven top factors for REC8 activation. Effect sizes and significance were calculated using a linear model with logit link. Significantly positive factors were BCL2, HOXB5, STRA8, and myr-AKT1 (*P* = 6 × 10^−7^, 8 × 10^−12^, 0.04, and 0.01, respectively). Error bars represent SEs of the coefficient estimates. (**D**) Screen of media and additives for REC8 activation (see Materials and Methods). Effect sizes and significance were calculated as above. *P* values for media additives (retinoic acid, AM580, PRT, RB3, and valproic acid) were 2 × 10^−25^, 7 × 10^−27^, 0.04, 9 × 10^−7^, and 4 × 10^−8^, respectively. (**E**) Effects of DNMT1i treatment on expression of DDX4, SYCP3, and REC8 reporters (*n* = 4). Error bars represent 95% confidence intervals. DNMT1i significantly up-regulated DDX4 (*P* = 0.01) and SYCP3 (*P* = 2 × 10^−4^).

We began by analyzing previously published scRNA-seq data of human fetal gonads, which contain a variety of cell types (*20*), including premeiotic *STRA8*^+^ (stimulated by retinoic acid gene 8) oogonia and fully meiotic oogonia/oocytes (Fig. 1A). We confirmed *REC8*, a meiosis-specific cohesin subunit, as a reliable marker for premeiotic cells, and *SYCP3*, a component of the SC, as a reliable marker for meiotic cells. Using previously constructed male and female DDX4-tdTomato reporter hiPSCs (*21*), we engineered dual reporter lines for DDX4-tdTomato/REC8-mGreenLantern (D4TR8G) and DDX4-tdTomato/SYCP3-mGreenLantern (D4TS3G) (fig. S1). We validated these lines by whole genome sequencing (*22*) and by CRISPRa and flow cytometry.

Next, we chose 78 candidate meiosis-promoting factors based on scRNA-seq analysis and previous literature (see Materials and Methods). We cloned these into a barcoded PiggyBac transposon plasmid for doxycycline-inducible expression. We integrated the library into the reporter hiPSCs, activated expression, and sorted reporter-positive cells after 7 days of induction (Fig. 1B). We tested low-copy and high-copy integration, as well as a variety of different culture media (see Materials and Methods). Comparing barcode frequencies between reporter-positive and unsorted populations, we found several factors consistently enriched in REC8^+^ cells (fig. S2), although results for the other reporters were noisy due to low cell yield.

### Optimization of REC8 activation

On the basis of the barcode enrichment results, we narrowed down our library to 16 factors and tested these individually for activation of REC8 and SYCP3 expression (fig. S3). BCL2 (a mitochondrial membrane stabilizer), HOXB5 [a Hox family transcription factor (TF)], and myr-AKT1 (a constitutively active form of protein kinase B) all slightly activated REC8, although no factors activated SYCP3. We next performed a combinatorial screen of seven factors and found that BCL2, HOXB5, STRA8, and myr-AKT1 all significantly promoted REC8 expression (Fig. 1C). Using these top four factors, we tested different media compositions, supplements, and induction times (Fig. 1D). The best-performing medium was APEL2, and retinoids (retinoic acid and AM580) significantly increased REC8 expression. The PRC1 inhibitors RB3 and PRT4164 caused a small increase in REC8 expression, but this was associated with extensive toxicity. Valproic acid was also toxic and significantly decreased REC8 expression. There was no significant change in REC8 expression between 6, 7, and 8 days of induction.

### DNMT1 inhibition up-regulates meiotic markers

Using the top factors and differentiation medium, we could induce REC8^+^ cells at nearly 100% efficiency, but cells still lacked SYCP3 expression. We reasoned that overexpressing meiosis-promoting factors might not be sufficient and that down-regulation of meiosis-inhibiting factors might be required. Therefore, we tested CRISPRi knockdown of 10 epigenetic factors. Knockdown of *DNMT1* resulted in a small up-regulation of SYCP3 (fig. S4). In previous work, we used a noncovalent DNMT1 inhibitor, GSK3484862, to erase DNA methylation and establish an oogonia-like epigenetic state (*21*). Treatment with this inhibitor resulted in a significant increase in expression of SYCP3, as well as the oogonia marker DDX4 (Fig. 1E).

### scRNA-seq screening identifies meiotic cells and associated factors

**Fig. 2.**
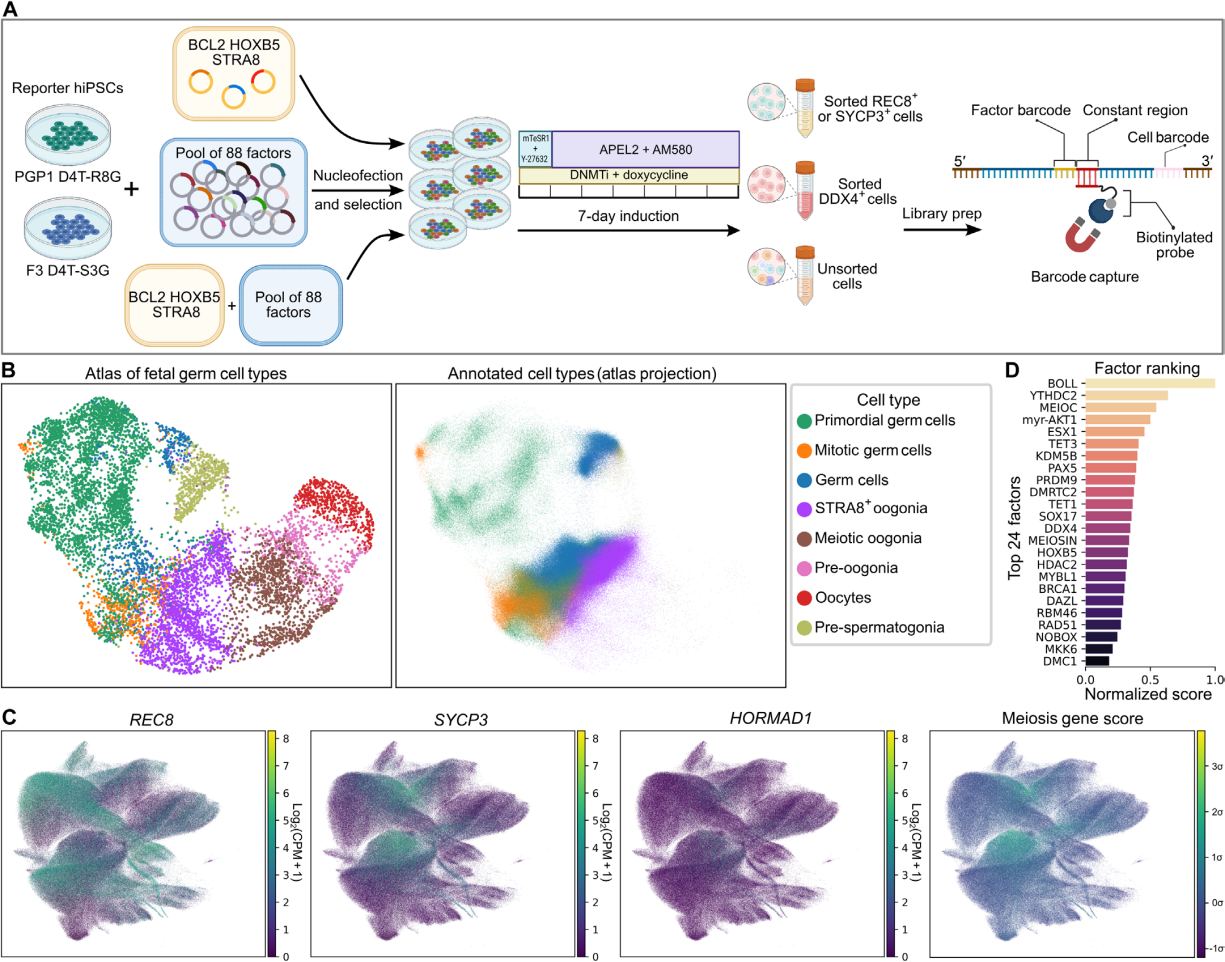
scRNA-seq identifies factors promoting meiotic cell identity. (**A**) scRNA-seq differentiation, library prep, and barcode capture (see Materials and Methods). (**B**) Cell type annotation based on the fetal germ cell atlas (*20*). The left is a UMAP plot of the fetal germ cell atlas, and the right is a projection of the hiPSC-derived cells onto the atlas UMAP manifold, with batch correction and cell type annotation performed using scanpy ingest. (**C**) UMAP plots showing expression of selected meiosis marker genes, as well as the meiosis gene score calculated from expression of 19 meiosis-specific genes, in hiPSC-derived cells in the screening experiment. The UMAP coordinates are specific to the hiPSC-derived cells and do not make use of the reference atlas. (**D**) Factor ranking based on barcode overrepresentation in cells with meiotic gene expression.

With DNMT1 inhibition, retinoid treatment, and overexpression of BCL2, HOXB5, and STRA8, we observed activation of REC8, SYCP3, and DDX4. However, we wanted to take a broader view of the gene expression in our cells and see whether expressing any additional factors could drive the cells closer to meiosis. Therefore, we generated iPSC populations containing integrated expression vectors for BCL2, HOXB5, and STRA8 under hygromycin selection, as well as a pool of 88 other candidate regulatory factors under puromycin selection (table S1). Following our induction protocol, we performed scRNA-seq on sorted reporter-positive cells as well as unsorted cells (Fig. 2A and see Materials and Methods).

We first investigated whether any meiotic cells were present in our samples. Leveraging the fetal gonad scRNA-seq dataset (*20*), we performed cell type annotation (Fig. 2B). Most of our cells were classified as premeiotic oogonia, and a small fraction of cells was annotated as fully meiotic. The proportion of these cells was greatest (0.8%) in sorted SYCP3^+^ samples. As another way of looking for meiotic cells, we constructed a score based on expression of meiosis-specific genes. In addition to *REC8* and *SYCP3*, the markers for which we sorted, we also observed expression of other essential meiosis genes, such as the axial element component *HORMAD1*, in a smaller fraction of cells (Fig. 2C). We chose a list of 19 meiosis-specific genes (table S2) and scored cells based on their expression. Of 646,493 cells in this preliminary screening experiment, 1276 cells had a gene score of >4σ, compared with an expected 20 cells assuming random gene expression.

Next, we asked which regulatory factors were responsible for generating these meiotic cells. We performed a hybridization-based capture to enrich our scRNA-seq library for barcode sequences and identified at least one expressed barcode in 91% of our cells. We then examined which factors were overrepresented in meiotic cells (defined using cell type annotation or gene scoring) versus premeiotic cells. We chose the top 24 for subsequent screening (Fig. 2D).

### Combinations of BCL2 and promeiotic factors are sufficient for inducing meiosis

We next expressed each of these 24 factors along with the previous top three (BCL2, HOXB5, and STRA8). After 7 days of induction, we monitored reporter activation and performed immunostaining for the meiosis markers HORMAD1 and SYCP3. We identified four factors, BOLL, MEIOC, MEIOSIN, and myr-AKT1, as the most promising (fig. S5A). We combined these four with the previous top three and repeated the experiment, this time analyzing a series of time points (7, 9, 13, and 16 days). Excitingly, at days 9 and 13 postinduction, we observed a few HORMAD1^+^ SYCP3^+^ γH2AX^+^ cells with zygotene-like filamentous staining (fig. S5B).

To narrow down which factors were responsible for inducing meiosis, we performed two rounds of combinatorial screening. In the first round, we tested 16 combinations of the initial set of seven factors (fig. S5, C, D, and E). No combination lacking BCL2 was able to induce zygotene cells as measured by HORMAD1 filament formation. Notably, we tested the combination of DAZL and BOLL overexpression which was previously reported to induce meiosis (*14*, *17*) but found that this was not sufficient (fig. S5F). BCL2/HOXB5/BOLL and BCL2/HOXB5/myr-AKT1/MEIOC were the best individual combinations. BCL2, HOXB5, and BOLL all induced a significant increase (three biological replicates each of 16 combinations; linear model, *P* = 3 × 10^−16^, 9 × 10^−3^, and 9 × 10^−3^, respectively), in the number of zygotene cells (fig. S5C). We found that STRA8 significantly decreased (*P* = 0.047) the zygotene score when overexpressed (fig. S5C).

**Fig. 3.**
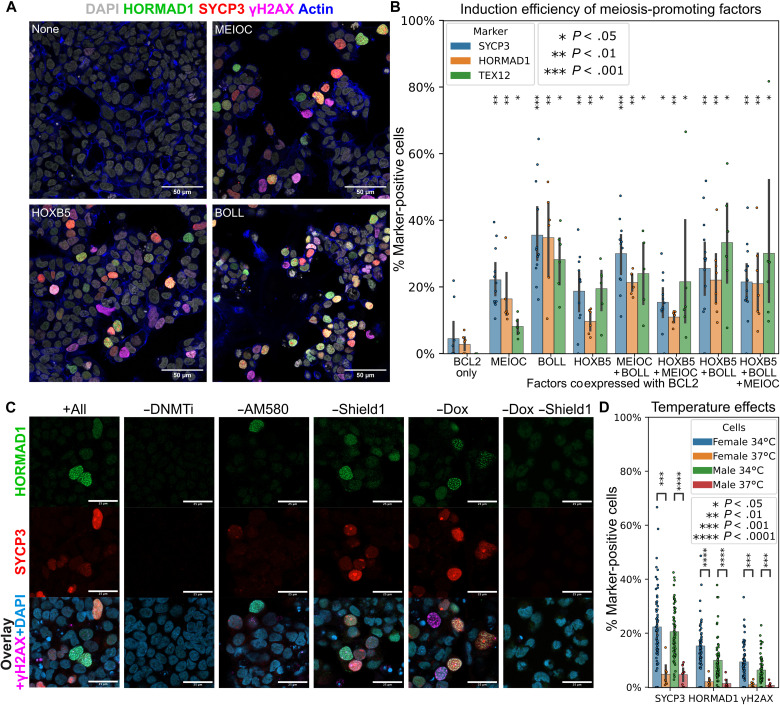
Optimization of meiosis induction. (**A**) Cells expressing constitutive BCL2 and Dox-inducible MEIOC, HOXB5, or BOLL were subjected to the meiosis induction protocol (see Materials and Methods) and stained for DNA [4′,6-diamidino-2-phenylindole (DAPI)], actin (phalloidin), HORMAD1, SYCP3, and γH2AX. Scale bars, 50 μm. (**B**) From the same experiment as (A), all eight possible combinations of the factors tested in three cell lines. Two images were analyzed per cell line and combination. *P* values represent comparisons to the BCL2-only control. Error bars represent 95% confidence intervals. (**C**) Cells expressing constitutive BCL2, Dox-inducible BOLL, and Shield1-inducible HOXB5 were subjected to the meiosis induction protocol omitting various factors (see Materials and Methods). Representative immunofluorescence images are shown. Scale bars, 25 μm. (**D**) Effects of performing meiosis induction in male and female hiPSCs at 34° or 37°C. Error bars represent 95% confidence intervals. In both sexes, the lower temperature significantly enhanced expression of SYCP3 (female: *P =* 2 × 10^−4^; male: *P =* 3 × 10^−5^), HORMAD1 (female: *P* = 3 × 10^−5^; male: *P =* 9 × 10^−5^), and γH2AX (female: *P* = 2 × 10^−4^; male: *P =* 1 × 10^−4^).

Last, we generated hiPSCs constitutively expressing BCL2 and inducibly expressing HOXB5, BOLL, and MEIOC, testing all eight possible combinations of these three factors. In addition to staining for meiosis markers (HORMAD1, SYCP3, and γH2AX), we also stained actin to show the overall morphology of the cells. We observed that each of the factors could induce meiosis when expressed with BCL2 (Fig. 3A). BOLL was the most efficient of the three factors we tested (Fig. 3B). In the BCL2-only control, we observed no HORMAD1^+^ cells and only a few occasional SYCP3^+^ cells.

### DNA demethylation and retinoid stimulation are required for efficient meiotic initiation

To further investigate the conditions necessary for meiotic initiation, we omitted different components of our protocol (Fig. 3C and fig. S6). Without DNMT1 inhibition, meiosis was completely blocked. However, DNMT1 inhibitor could be withdrawn after the first 5 days without negatively affecting results. Omitting the retinoid AM580 resulted in fewer meiotic cells, but some were still present. If AM580 treatment was started later than day 5, then results were similarly poor. Last, using orthogonal induction systems [doxycycline (Dox) and Shield1] for BOLL and HOXB5 expression, we confirmed that the expression of at least one of these factors was required for inducing meiosis.

### Lower temperatures enhance meiotic induction

In males, meiosis takes place in the adult testes, which are cooler than the rest of the body. Previous studies indicated that male meiosis is less efficient at 37°C (*10*). Therefore, we tested our meiosis induction protocol at 34°C versus 37°C using male and female hiPSCs. Meiosis induction was significantly enhanced at 34°C in not only male but also female cells (Fig. 3D and fig. S7A). We tested 34°C starting at either day 1 or day 3 of the induction protocol and found that both worked equally well. Monitoring the cells for up to 21 days, we saw that cell viability declined past day 16. We noticed that REC8-mGreenLantern fluorescence was much weaker at 34°C compared to 37°C, whereas SYCP3-mGreenLantern and DAZL-mGreenLantern were equally bright (fig. S7B).

### Meiosis induction directly generates meiotic cells without transitioning through PGC-like intermediates

**Fig. 4.**
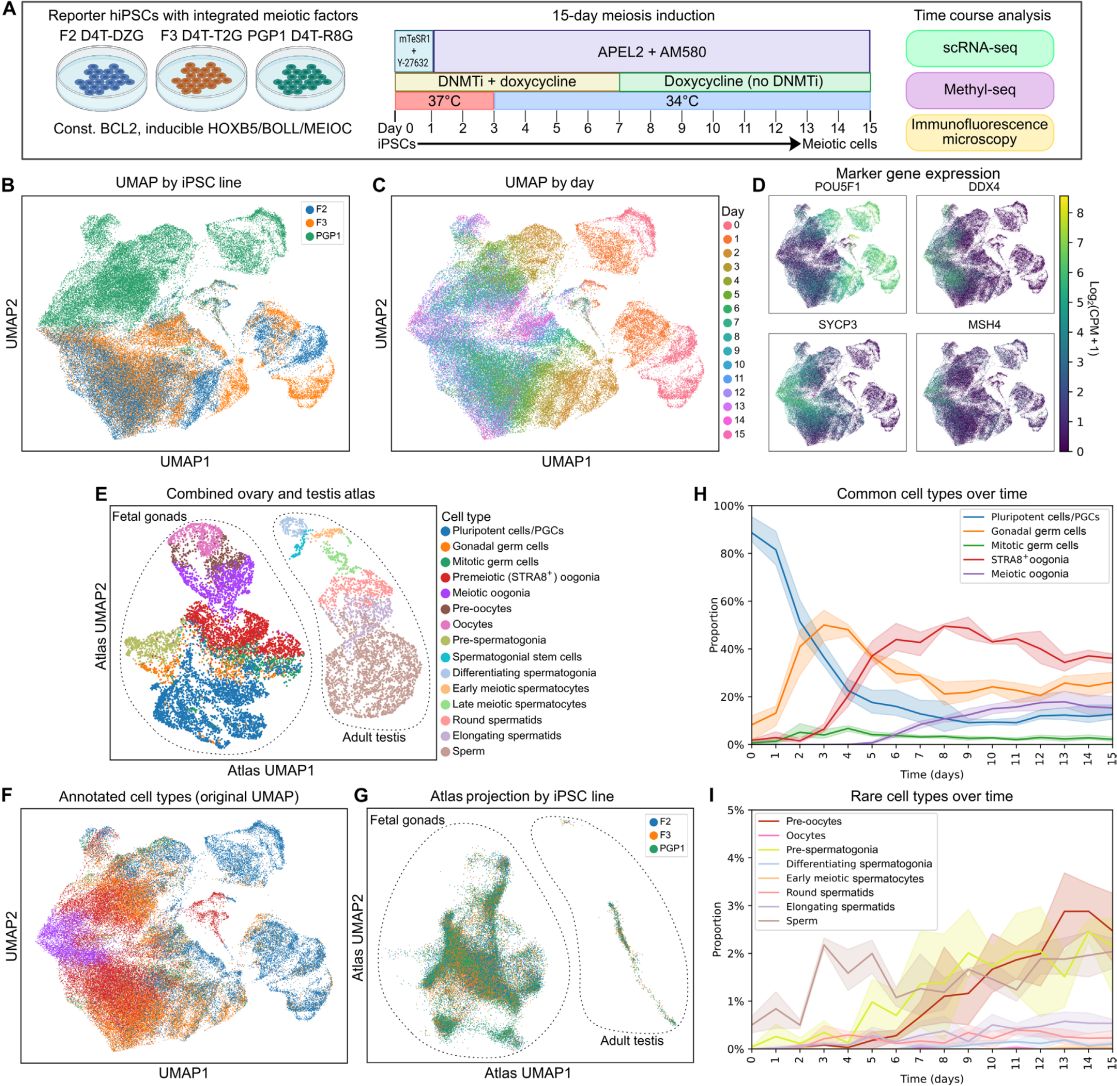
Time course scRNA-seq analysis of meiosis induction. (**A**) Overview of the meiosis induction protocol. Data from this experiment are shown in Fig. 4 (scRNA-seq), Fig. 5 (immunofluorescence), and fig. S9 (methyl-seq). (**B**) UMAP plot, colored by hiPSC line. PGP1 is male; F2 and F3 are female. (**C**) UMAP plot, colored by day of sample collection. (**D**) UMAP plots colored by expression of marker genes for pluripotency (*POU5F1*), oogonia/gonocytes (*DDX4*), meiosis (*SYCP3*), and recombination (*MSH4*). (**E**) Cell types present in the combined ovary and testis reference atlas. (**F**) UMAP plot of annotated cell types over 15 days of meiosis induction. (**G**) iPSC-derived cells projected onto the atlas UMAP, colored by cell line. (**H**) Proportions of common (>5% abundance) cell types over time. (**I**) Proportions of rare (<5% abundance) cell types over time.

We next investigated the progression of gene expression over time in our optimized meiosis induction protocol. Using three hiPSC lines (two females and one male), we performed Parse split pool–based scRNA-seq on a total of 16 time points per line, every 24 hours from the beginning of our induction protocol (day 0; hiPSCs) through day 15 (Fig. 4A). Our postfiltering dataset comprised a total of 69,018 cells from one male (PGP1) and two female (F2 and F3) cell lines and 16 time points spanning days 0 to 15. We first performed dimensionality reduction (Fig. 4, B and C) and examined marker gene expression (Fig. 4D and fig. S8). Cells from the two female lines overlapped, but the male cells were largely separate (Fig. 4B). However, at later time points, the male and female lineages converged (Fig. 4C). Expression of the pluripotency marker *POU5F1* was initially high (Fig. 4D) but quickly declined and reached low levels by day 6. At intermediate time points, cells began to express gonadal germ cell markers, including *DDX4* (Fig. 4D), *DAZL*, *MAEL*, *STK31*, and *MAGE* and *PIWI* family genes (fig. S8). Cells also expressed marker genes for meiosis, including all components of the SC. *REC8* was one of the earliest meiosis genes expressed. By days 12 to 15, a subset of cells strongly expressed recombination markers such as *MSH4* (Fig. 4D and fig. S8). Markers for gametes (oocytes and sperm) were not highly expressed. In addition, we noted that *SPO11*, the nuclease required to initiate meiotic recombination, was expressed in only a small fraction of cells.

To compare our cells with in vivo germ cells, we constructed a scRNA-seq atlas by combining data from human fetal gonads (containing female meiotic cells) and adult testis (containing male meiotic cells) from two previously published atlases, both of which used 10× droplet-based scRNA-seq (Fig. 4E) (*20*, *23*). We projected our cells onto the combined atlas and performed cell type annotation (Fig. 4, F and G). This analysis showed that our cells were more similar to fetal ovarian cells, although a few cells were classified as adult testicular cells. When projected onto the atlas Uniform Manifold Approximation and Projection (UMAP), cells from all three iPSC lines overlapped, and there was no clear distinction between male and female lines (Fig. 4G). As an additional comparison, we computed the Spearman correlation of pseudobulk gene expression for each cell type in our dataset and the atlas. Cells annotated as meiotic oogonia in our dataset were most similar to meiotic oogonia in the reference atlas (fig. S9A), and the correlation coefficient (0.68) was within the range reported for identical cell types sequenced using Parse versus 10× modalities (*24*).

We next examined the proportions of each cell type over time, plotting common (>5% abundance) and rare (<5% abundance) cell types separately (Fig. 4, H and I). Although the cells at early time points were largely annotated as PGCs, this reflects expression of marker genes such as *POU5F1* and *NANOG* which are shared between pluripotent cells and PGCs rather than a bona fide PGC-like state. Our cells lacked expression of definitive PGC marker genes including *NANOS3*, *PRDM1*, *SOX17*, and *TFAP2C* (fig. S8). Despite skipping over the PGC state, our cells transitioned through gonadal germ cell and oogonia-like states before entering meiosis (Fig. 4H). Fully meiotic cells were first present at day 6, with the proportion increasing through day 12. The percentage of meiotic cells ranged between 16 and 22% among the three cell lines tested. At later time points, some cells were classified as diplotene arrested (pre-oocytes or oocytes) or postmeiotic (round spermatids, elongating spermatids, or sperm). The proportion of these cell types increased over time and reached a maximum on day 13 (Fig. 4I). However, these late-stage cells, especially those annotated as “sperm,” may not fully reflect their in vivo counterparts. In particular, we did not observe expression of sperm-specific marker genes (fig. S8).

### DNA methylation is rapidly but incompletely erased during meiosis induction

Because a crucial part of our protocol involves treating cells with a DNMT1 inhibitor, we next investigated to what extent the cells erased their DNA methylation over time. In a pilot experiment, we measured global DNA methylation using low-coverage methyl-seq of samples from days 0 to 15 (Fig. 4A and fig. S10A). As expected, day 0 cells (hiPSCs) had 80 to 85% overall methylation. Methylation levels quickly declined for the first 2 days but reached a plateau of 27 to 34%, where they remained for the rest of the protocol. Despite DNMT1 inhibitor being withdrawn on day 7, cells did not regain DNA methylation afterward.

Hypothesizing that the subset of cells with oogonia marker expression had lower DNA methylation than the others, we performed an additional methyl-seq experiment at 11× to 14× genome-wide coverage on cells from the meiosis induction. We analyzed hiPSCs, day 2 cells, and sorted DDX4-tdTomato–positive and DDX4-tdTomato–negative cells from days 5, 10, and 15. We observed a similar trend in methylation over time, but contrary to our hypothesis, DDX4^+^ cells did not have significantly less genome-wide methylation than DDX4^−^ cells (paired two-tailed *t* test, *n* = 18, *P* = 0.30). However, DDX4^+^ cells had significantly lower methylation at the promoters for key oogonia and meiosis genes including *DDX4*, *DAZL*, and *SPO11* (*P* = 0.03, 0.01, and 0.02; fig. S10B). These genes were all characterized by a high degree of promoter methylation (80 to 100%) in hiPSCs. By contrast, the *REC8* promoter was only 20% methylated in hiPSCs, and although its methylation declined to near-zero in both DDX4^+^ and DDX4^−^ cells, this did not significantly differ between the two groups (*P* = 0.32). In general, most promoters had their methylation erased in both DDX4^+^ and DDX4^−^ cells (fig. S10C). Likewise, the median imprinting control region methylation decreased from 84% in hiPSCs to 14% by day 15 (fig. S10D). Results for feature methylation and genome-wide differentially methylated regions are provided as file S1. Overall, our meiosis induction protocol quickly erases DNA methylation, including at promoters of key regulatory genes as well as imprinting control regions. However, the erasure is not fully complete, likely because it depends on proliferation in the absence of DNMT1 activity and cells cease to proliferate upon entering meiosis.

### Single-cell multiomic analysis reveals TF regulon activity in meiotic cells

To further explore the interaction between epigenetic state and gene expression in our meiotic cells, we performed single-cell multiomics [Assay for Transposase-Accessible Chromatin (ATAC) + RNA] on isolated nuclei from day 15 of meiosis induction in three cell lines (two females and one male). We first examined the overall chromatin accessibility in relation to genomic features including promoters, imprints, cytosine-phosphate-guanine (CpG) islands, and transposable elements (TEs). We observed enrichment of ATAC peaks in promoter regions (fig. S11A) and a bimodal distribution of promoter accessibility (fig. S11B). Compared to the genome-wide average, promoters, imprint control regions, and CpG islands were more accessible, whereas TEs were less accessible (table S7).

To annotate cell types, we used scanpy ingest on our RNA data with the human fetal germ cell atlas (*20*) as a reference (fig. S11C). For subsequent analysis, we defined meiotic cells as both oogonia_meiotic and oogonia_STRA8 cell types. We confirmed the presence of known meiotic genes, including *DMC1*, *SYCP1*, and *SYCP3*, on a UMAP chart (fig S11D). As with our Parse-based scRNA-seq, we observed only a small amount of *SPO11* expression. Comparisons with in vivo data indicate that our in vitro–derived meiotic cells capture some, but not all, features of bona fide meiotic oogonia (*20*). RNA-based joint clustering shows similar patterns in the second UMAP dimension (fig. S11E), although the first UMAP dimension separates cells by study. A Spearman RNA correlation pseudobulk analysis shows high values in the 80 to 90% range between corresponding cell types (fig. S9B). The ATAC joint clustering UMAP also shows a similar pattern in the second UMAP dimension (fig. S11F). The same joint ATAC UMAP, plotted after using Scanpy ingest with the in vivo ATAC atlas as a reference to reduce batch effects, shows even stronger similarity in accessible regions for corresponding cell types, supporting their biological similarity (fig. S11G).

To examine chromatin accessibility differences between our meiotic cells and those from the reference atlas, we performed a DESeq2 analysis of consensus peak ATAC regions (fig. S11H). Overall, we found that 1.47% of regions in our meiotic cells were significantly less accessible than in vivo, and 0.87% were significantly more accessible. The most significantly less accessible gene was *TPTE*, a phosphatase and tensin homolog (*PTEN*)-like tyrosine phosphatase expressed in meiotic oogonia in vivo (*20*). We performed gene ontology (GO) term enrichment analysis for differentially accessible regions (DARs). No GO terms were significantly enriched for regions that were more accessible than in vivo. For regions that were less accessible than in vivo, we found several enriched GO terms, including “meiotic cell cycle checkpoint signaling,” “outer mitochondrial membrane organization,” and “mRNA destabilization.” A full list of DARs and GO terms is provided in table S8.

To characterize potential regulatory circuits in our in vitro–derived meiotic cells, we used SCENIC+, an inference framework which integrates chromatin accessibility with gene expression to detect enriched TF motifs and link them to target genes (*25*). We first examined the ATAC data for TF motifs enriched in meiotic cells versus nonmeiotic cells (fig. S11G and table S8). We observed strong enrichment for several zinc finger and E2F family factors, which have GC-rich binding motifs. This could be related to the role of CpG methylation in suppressing expression of meiotic genes in nonmeiotic cells. In addition, E2F6 is a component of the PRC1.6 complex, which is known to suppress meiotic genes (*26*), which may explain why regions with this binding motif are less accessible in nonmeiotic cells. Integrating RNA data with ATAC data, we computed TF regulon activity scores for meiotic versus nonmeiotic cells. We identified several known promeiotic TFs, including *ZNF541* (*27*), *PRDM9* (*28*), and *E2F1* (*29*), as well as the recombination factor *BRCA1*. Other top factors (including *IKZF3*, *HEXS1*, *DMRTC2*, and *ZNF280B*) have not been previously studied in context of meiosis but are expressed in meiotic cells (*20*) and may represent drivers of meiosis.

### Meiotic cells progress through leptonema and zygonema but only rarely reach pachynema

**Fig. 5.**
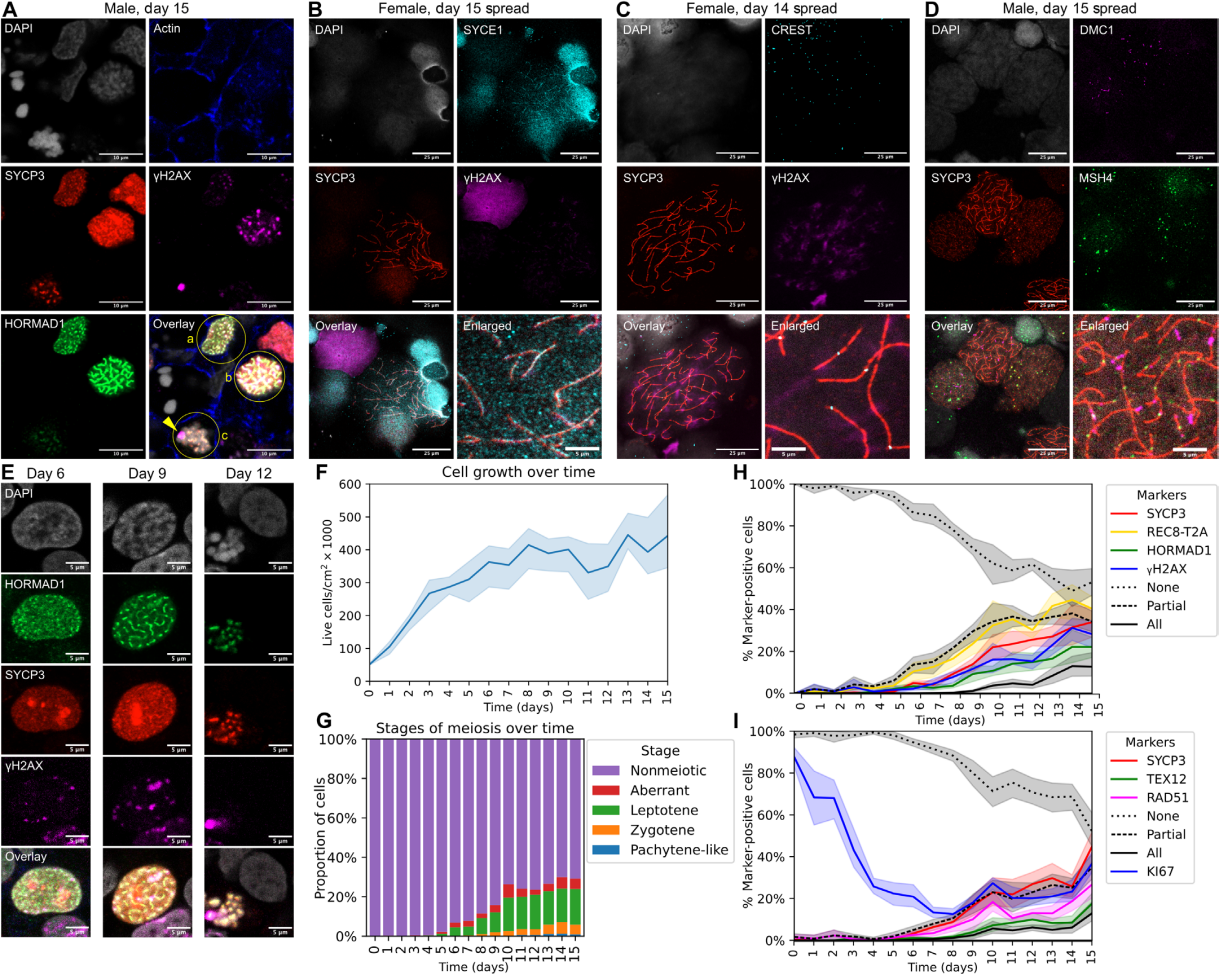
Stages of meiosis and their progression over time. (**A**) Immunostaining of day 15 male meiotic cells, labeled as (a) leptotene, (b) zygotene, and (c) pachytene-like. A γH2AX-positive sex body, labeled with an arrowhead, is visible on the periphery of the pachytene-like nucleus. Scale bars, 10 μm. (**B**) Chromosome spread of female meiotic cells stained for DNA (DAPI), SYCP3, SYCE1, and γH2AX. Scale bars, 25 μm. (**C**) Chromosome spread of female meiotic cells stained for DNA (DAPI), SYCP3, centromeres (CREST), and γH2AX. Scale bars, 25 μm. (**D**) Chromosome spreads of male meiotic cells stained for DNA (DAPI), recombination markers (DMC1 and MSH4), and SYCP3. Scale bars, 25 μm. (**E**) Representative images of nuclei at different time points during meiosis induction from male hiPSCs. Scale bars, 5 μm. (**F**) Cell growth over 15 days of meiosis induction (two female and one male hiPSC line; protocol shown in Fig. 4A). (**G**) Classification of stages of meiosis, based on staining for DNA (DAPI), SYCP3, REC8, HORMAD1, and γH2AX (two images analyzed per line per time point). REC8 expression was measured by staining for the T2A linker peptide. (**H**) Proportions of cells expressing meiotic markers (SYCP3, REC8, HORMAD1, and γH2AX) over 15 days of induction, as well as proportions of cells expressing all (4), partial (1 to 3), or no markers (two images analyzed per line per time point). (**I**) Proportions of cells expressing meiotic markers (SYCP3, TEX12, and RAD51) and KI67 over 15 days of induction, as well as proportions of cells expressing all (3), partial (1 to 2), or no meiotic markers (two images analyzed per line per time point).

To analyze which stages of meiosis were present in our cells, we performed costaining for HORMAD1, which marks the chromosome axis and is removed from synapsed chromosomes during pachynema, SYCP3, which marks the lateral elements of the SC, and γH2AX, which marks recombination-related DNA damage in leptonema and zygonema, and the sex body (unsynapsed XY chromosomes) of male cells in pachynema and diplonema (*18*). By day 12 of our induction protocol, three stages of meiosis (leptonema, zygonema, and pachynema) were visible. A representative image of these three stages is shown in Fig. 5A. The leptotene cell (Fig. 5A, overlay, labeled a) has diffuse HORMAD1 and SYCP3 expression and a faint γH2AX signal. The zygotene cell (Fig. 5A, overlay, labeled b) has filamentous HORMAD1 and SYCP3 and stronger γH2AX. In addition, the chromosomes are starting to compact, as seen in the 4′,6-diamidino-2-phenylindole (DAPI) channel. The pachytene-like cell (Fig. 5A, overlay, labeled c) has fully compacted chromosomes, associated with SYCP3 staining. HORMAD1 staining is much weaker, indicating that synapsis has occurred, and a γH2AX^+^ putative sex body (labeled with an arrowhead) is visible on the nuclear periphery. Notably, we observed these putative sex bodies only in male cells. The degree of chromosome compaction and SYCP3 staining is morphologically similar to pachytene-stage primate oocytes (*30*). A three-dimensional z-stack of this image is provided as movie S1.

Meiotic cells expressed cytoplasmic DDX4, an RNA binding protein specifically expressed in germ cells in vivo starting at the oogonia or spermatogonia stage (fig. S12A) (*31*), and TEX14, a protein that forms intercellular bridges between meiotic germ cells in vivo (fig. S12B) (*32*), although the TEX14 expression pattern was not organized into bridges. We also observed nuclear foci of the recombination markers RAD51 recombinase (fig. S12A), mutS homolog 4 (MSH4; fig. S12, B and C), and mutL homolog 1 (MLH1; fig. S12C).

We additionally performed chromosome spreads as another way of examining stages of meiosis. Here, we observed only leptotene and zygotene cells (Fig. 5, B, C, and D). The SC central element protein SYCE1 was localized to chromosome axes (marked with SYCP3) in some cells, but it was not present along their entire length (Fig. 5B). Although chromosome axes formed on all 46 chromosomes as seen by staining for SYCP3 and centromeres (Fig. 5C), we did not observe any cells in which full synapsis had taken place. We observed puncta of the meiotic recombination markers MSH4 and DMC1 along the chromosome axes (Fig. 5D), showing that double-strand breaks had been processed into recombination intermediates. Overall, our protocol can reliably reach the first two stages of meiosis I. Cells form chromosome axes and initiate recombination but do not efficiently establish synapsis and progress to pachynema.

### Meiotic progression over 15 days of induction

Using three hiPSC lines (two females and one male), we performed immunofluorescence imaging on a total of 16 time points per line during the same experiment as the scRNA-seq time course (Fig. 4A). We performed two sets of stains to monitor expression and colocalization of marker proteins: REC8-T2A/SYCP3/HORMAD1/γH2AX and KI67/SYCP3/TEX12/RAD51. These markers are specific to meiosis, with the exception of KI67, which is a marker of proliferating (mitotic) cells and meiotic cells (*33*).

In our time course data, we observed a progression of meiotic cells through leptotene (diffuse expression of SYCP3, HORMAD1, and γH2AX), zygotene (filamentous HORMAD1 and SYCP3), and pachytene-like (condensed chromosomes, thicker SYCP3 filaments, weaker HORMAD1, and γH2AX absent or restricted to a putative sex body) stages (Fig. 5E). We counted the number of live cells at each time point. The cells proliferated ~8-fold over the first week of the protocol, but the number remained stable after day 8 (Fig. 5F). To quantify the prevalence of different stages of meiosis, we manually annotated 1707 meiotic nuclei based on staining for HORMAD1, SYCP3, and γH2AX (Fig. 5E; see Materials and Methods) and applied it to our imaging data (Fig. 5G). Leptotene cells were first seen at day 6, with the proportion increasing through day 10. Zygotene and pachytene-like cells were observed at later time points, and there were also a small number of cells classified as aberrant due to discordant marker expression (for example, apoptotic γH2AX^+^ cells). By day 15, the overall proportions of stages were 18.2% leptotene, 4.9% zygotene, 0.9% pachytene-like, 5.2% aberrant, and the remainder (70.8%) nonmeiotic.

In accordance with the early expression of *REC8* in scRNA-seq (fig. S8), REC8-T2A was the first of the meiotic marker proteins to be expressed, starting at day 6 and continuously increasing through day 11 (Fig. 5H). HORMAD1, SYCP3, γH2AX, and RAD51 expression followed similar trajectories, starting around days 7 to 8 and increasing through day 15. TEX12, which is required for chromosome synapsis in zygonema and pachynema (*34*), was the last marker to be expressed, starting around day 9 and increasing thereafter (Fig. 5I). The kinetics were similar in male and female hiPSC lines. By day 15, an average of 13% (range: 6 to 28%, *n* = 6 biological replicates) of cells was quadruple-positive for all markers in the first stain combination (REC8/HORMAD1/SYCP3/γH2AX), with an additional 34% (range: 26 to 43%) of cells being positive for at least one marker but not all of them. In the second stain combination, an average of 13% (range: 9 to 25%, *n* = 6 biological replicates) were triple-positive for all meiotic markers (SYCP3/TEX12/RAD51), and an additional 35% (range: 25 to 48%) were positive for at least one marker but not all of them. At the beginning of the protocol, nearly all cells were positive for KI67, a marker of proliferating cells and meiotic cells (*33*). As cell proliferation slowed, KI67 decreased from days 0 to 7 but remained expressed in meiotic cells (Fig. 5I). Representative images over a series of time points are shown in fig. S13, and a full set of images is available in an online repository (see Data and materials availability).

### Fluorescent reporters show chromosome synapsis and enable imaging of meiosis in live human cells

In other species such as mice, imaging of the SC in live cells has provided important insights into meiosis (*35*). Despite the low rate of synapsis in our protocol, we set out to perform similar imaging in human cells. We constructed mStayGold-tagged knock-in reporter lines for SIX6OS1 (also known as C14orf39) and TEX12, both of which are SC central element components (*36*, *37*).

**Fig. 6.**
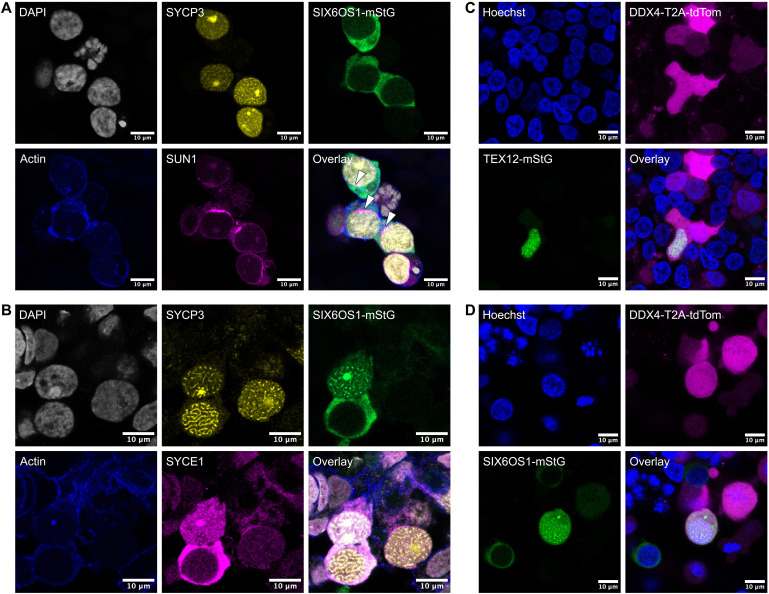
Fluorescence microscopy shows features of meiosis. (**A**) Imaging of SIX6OS1-mStayGold reporter in fixed cells, along with staining for DNA (DAPI), SYCP3, actin (phalloidin), and SUN1. White arrowheads mark regions of the nuclear membrane enriched for SUN1. (**B**) Imaging of SIX6OS1-mStayGold reporter in fixed cells, along with staining for DNA (DAPI), SYCP3, actin (phalloidin), and SYCE1. SIX6OS1 and SYCE1 colocalize and are present either in the cytoplasm (bottom cell) or as nuclear filaments (middle cell). (**C**) Imaging of TEX12-mStayGold and DDX4-T2A-tdTomato reporters in live cells, along with staining for DNA (Hoechst). TEX12 forms nuclear filaments. (**D**) Imaging of SIX6OS1-mStayGold and DDX4-T2A-tdTomato reporters in live cells, along with staining for DNA (Hoechst). SIX6OS1 is present either in the cytoplasm or as nuclear filaments. Scale bars, 10 μm.

Performing our meiosis induction protocol, we observed reporter expression beginning at day 9. Initially, SIX6OS1-mStayGold was present only in the cytoplasm (Fig. 6A). Staining of fixed cells revealed that SIX6OS1 was expressed in a subset of SYCP3^+^ meiotic cells. We additionally stained for SUN1, a nuclear envelope protein which binds telomeres during meiosis (*38*), and saw that it had concentrated to one spot of the nuclear envelope in SIX6OS1^+^ cells, reflecting the process of meiotic telomere bouquet formation (*38*, *39*). At later time points, SIX6OS1 had relocated to the nucleus in some cells. Using day 15 fixed cells, we stained for SYCE1, another central element protein which directly binds SIX6OS1 in vivo (*36*), and observed that it colocalized with SIX6OS1 (Fig. 6B). The presence of nuclear filaments staining positive for SYCP3, SIX6OS1, and SYCE1 indicated SC central element formation and at least partial chromosome synapsis. Last, we imaged TEX12 and SIX6OS1 reporters in live human cells, also observing the DDX4-T2A-tdTomato reporter. We observed nuclear filaments formed by both TEX12 (Fig. 6C) and SIX6OS1 (Fig. 6D) in a subset of the DDX4^+^ cells. This live imaging proof of concept shows the value of an in vitro protocol for inducing meiosis in human cells.

## DISCUSSION

Here, we report a reliable and rapid protocol for inducing meiosis in male and female human cells. Our method relies on overexpressing BCL2 and at least one meiosis-promoting factor. We identified HOXB5, BOLL, and MEIOC as able to perform this role. Of these, BOLL and MEIOC were previously reported as promeiotic (*14*, *40*). HOXB5 was known to be expressed in fetal oogonia (*20*), but its role in meiosis was not previously studied. The most likely role of BCL2 in our protocol is to prevent apoptosis resulting from DNA double-strand breaks during leptonema (*41*). However, it is possible that BCL2 plays an additional role, as BCL2 alone was sufficient to up-regulate REC8 (fig. S3B). In accordance with previous studies in mice (*2*, *4*, *10*), we additionally show that DNA demethylation is required for meiotic entry and that retinoid treatment and lower temperatures increase the efficiency. Our induction protocol uses fully chemically defined culture media, enabling investigation of human meiosis under controlled conditions.

Comparing the gene expression of our cells to in vivo meiotic cells, we find that meiotic cells induced from both male and female hiPSCs are more similar to meiotic oogonia in vivo, although a small fraction (<5%) are similar to meiotic spermatocytes. Although our cells express oogonia/gonocyte markers, they do not transition through a PGC-like stage before meiotic entry, suggesting that this stage is not required for meiosis. The greater similarity of our cells to female (rather than male) meiotic cells may result from their epigenetic status, since in the female germ line, meiosis occurs in hypomethylated oogonia, whereas in the male germline, DNA methylation is reestablished before meiosis (*1*).

The primary limitation of our method is its low efficiency in producing pachytene and later-stage cells. Notably, only a small fraction of our cells expresses *SPO11*, and although the presence of γH2AX and RAD51 indicates that at least some double-strand breaks are formed, the low rate of chromosome synapsis may reflect defects related to recombination. We are now using polyclonal populations of iPSCs with randomly integrated expression vectors, and switching to a system that allows precise control of transgene expression levels may improve results. Furthermore, a more complete erasure of DNA methylation may benefit expression of meiotic genes. Although the genome-wide methylation level decreased from ~80% in hiPSCs to ~30% in our cells (fig. S10), this is still higher than the levels observed in premeiotic oogonia in vivo, which are as low as 4% (*1*). Similarly, we identified genomic regions in our meiotic cells with lower chromatin accessibility than in vivo (fig. S11H), with related GO terms including meiotic checkpoint signaling. In addition, in cultured mouse spermatogonia, meiosis has lower efficiency and fidelity compared with meiosis in vivo (*10*), suggesting an important role for the gonadal niche. Thus, integrating meiotic cells into recently developed ovarian organoid systems may be beneficial (*42*, *43*). Despite its limitations, our current method is easily scalable and produces zygotene meiotic cells in a relatively short time (13 to 15 days), similar to the known duration of human meiosis (*44*).

The ability to induce meiosis using human cells in vitro will unlock opportunities for science and medicine. Two examples include screening candidate male contraceptives and using gene-edited hiPSCs to investigate effects of genetic variants that have been hypothesized to cause human infertility (*45*). Future developments could allow the generation of genetic crosses between different human cell lines or the production of human gametes in vitro, which, after regulatory approval and societal acceptance (*46*), has the potential to treat most cases of infertility and expand reproductive autonomy. We believe that our method of inducing meiosis will greatly benefit research into this important reproductive process.

## MATERIALS AND METHODS

### Experimental design

#### iPSC culture

Human iPSC lines [American Type Culture Collection (ATCC)–BXS0115, referred to as F2, ATCC-BXS0116, referred to as F3, and PGP1] were cultured in mTeSR Plus medium (STEMCELL Technologies) on standard polystyrene plates coated with stem cell–qualified Matrigel (Corning). hiPSCs were grown in a 37°C 5% CO2 incubator. Passaging was performed by brief (3 to 5 min) treatment with 0.5 mM EDTA and 0.25× TrypLE Express (Gibco) in phosphate-buffered saline (PBS), followed by pipetting to break the colonies into small clumps. Cells were treated with 10 μM Y-27632 for 24 hours after each passage. Cells were tested every 3 months for mycoplasma using the ATCC Universal Mycoplasma Detection Kit. All cells tested negative. For experiments requiring single-cell dissociation and counting, cells were harvested with Accutase and counted using trypan blue staining with a Countess II automated cell counter (Thermo Fisher Scientific). Research on human cell lines was approved by the Harvard Embryonic Stem Cell Research Oversight Committee (project #21-111, “Multimodal Investigation of Human In Vitro Oogenesis”) and the Broad Institute Office of Research Subjects Protection (#NHSR-8350). This study was not classified as human subject research because only deidentified human iPSC lines from commercial sources were used, and no new iPSCs were derived as part of this study.

#### Generation and verification of reporter lines

Knock-in donor plasmids targeting *REC8* and *SYCP3* were constructed by Gibson assembly of 5′ and 3′ homology arms, an insert containing a fluorescent marker joined to the gene of interest by a T2A linker, and a plasmid backbone containing an MC1-DTA marker to select against random integration. Single guide RNA (sgRNA) oligos were cloned into pX330 (Addgene, #42230), which also expresses Cas9. Oligo sequences are provided in table S3. All plasmids are available on Addgene.

Knock-in electroporations were performed as previously described (*42*). In summary, 1 μg of donor plasmid and 1 μg of sgRNA/Cas9 plasmid were coelectroporated into 200,000 hiPSCs using a Lonza Nucleofector with 20 μl of P3 solution and pulse setting CA-137. Colonies were picked after selection and genotyped by polymerase chain reaction (PCR). Successful knock-in, the absence of off-target edits, and euploidy of the reporter lines were all confirmed by whole genome sequencing (Novogene, 10× coverage) and SeqVerify computational analysis (*22*). REC8 and DDX4 reporter lines were homozygous for the reporter allele, and SYCP3, SIX6OS1, and TEX12 reporter lines were heterozygous with an intact nonedited allele. Low-passage cells were cryopreserved using CryoStor CS10 (STEMCELL Technologies) and banked for future use.

Functional validation of the reporter alleles was performed by CRISPRa, except for SIX6OS1 and TEX12 reporters which were validated via meiosis induction. For each allele, three CRISPRa plasmids were constructed, each containing a sgRNA targeting the promoter as well as a doxycycline-inducible expression cassette for dCas9-VPR (*47*). Equimolar mixtures of plasmids (1 μg in total) were electroporated into 200,000 hiPSCs using a Lonza Nucleofector with 20 μl of P3 solution and pulse setting CA-137. After 2 days, cells were harvested with Accutase and analyzed by flow cytometry (fig. S1).

#### Identification of candidate meiosis-promoting factors

We obtained a human fetal germ cell scRNA-seq dataset from Garcia-Alonso *et al.* (*20*) (https://reproductivecellatlas.org/gonads.html). We performed pySCENIC analysis (*48*), which involves inferring a gene regulatory network, finding TF regulons based on known binding motifs, and ranking regulon activity in each cell type. We chose 21 TFs to screen based on high activity in STRA8^+^ and/or meiotic oogonia. Because regulon analysis ignores non-TF factors, including RNA binding proteins, we selected 18 additional factors to screen based on a gene regulatory network analysis. In this analysis, we found differentially expressed genes between meiotic oogonia and all other cell types and then calculated which genes in the regulatory network were upstream of the differentially expressed genes, multiplying network edge weights by differential expression fold changes to calculate a weighted score. We included a further 12 factors based on literature reports of promeiotic function. Last, we included 27 factors from the Cancer Pathways Open Reading Frames library (*49*), which contains modulators of several common cellular signaling pathways. Our initial library for barcode enrichment screening contained 78 factors. For scRNA-seq screening, we included an additional 10 factors involved in epigenetics and signal transduction, bringing the total to 88. A full list of the factors in our library is provided in table S1.

#### Plasmid library construction and PiggyBac transposon integration

Expression plasmids for 88 candidate regulatory factors were constructed by MegaGate cloning (*50*) into a barcoded PiggyBac destination vector containing a doxycycline-inducible promoter (*47*), as well as a puromycin selection marker. Barcodes and transgene sequences were verified by Sanger sequencing. For later experiments, an alternative version of the plasmid lacking the barcode and containing a hygromycin selection marker was constructed for top candidate factors. For constitutive BCL2 expression, a version of the plasmid was constructed with an EF1a promoter instead of a doxycycline-inducible promoter. All expression plasmids are available on Addgene.

Plasmids were pooled and coelectroporated into iPSCs along with a PiggyBac transposase expression plasmid (Systems Bioscience), using a Lonza Nucleofector with pulse setting CA-137. For medium-copy (three to five per cell) integration, 5 fmol of pooled library and 500 ng of transposase plasmid were used per 200,000 hiPSCs and 20 μl of P3. For high-copy (10 to 50 per cell) integration, 50 fmol of pooled library was used instead. Average integration numbers were previously characterized for the same transposons used in this study (*47*) but not evaluated directly. Selection was performed with puromycin (400 ng/ml) and/or hygromycin (50 μg/ml) beginning 2 days after nucleofection and continuing for at least 5 additional days before subsequent experiments.

#### Flow cytometry

Flow cytometry was performed on a BD LSR Fortessa instrument. Cell sorting was performed on a Sony SH800S sorter using a 100-μm chip. Compensation controls were acquired using cells in which mGreenLantern and tdTomato reporters had been activated by CRISPRa. DAPI (100 ng/ml) was used to stain dead cells for exclusion. Data analysis was performed using the Cytoflow python package (v. 1.0.0) (*51*). Representative gating is shown in fig. S3A.

#### Immunofluorescence microscopy

Cells were cultured on Matrigel-coated ibidi dishes (8-well, catalog no. 80826; or 96-well, catalog no. 89626). For eight-well dishes, 100 μl of staining solutions and 200 μl of wash solutions were used per well. For 96-well dishes, 50 μl of staining solutions and 100 μl of wash solutions were used per well. All steps except primary antibody incubation were performed at room temperature.

Cells were washed with PBS and fixed with 4% paraformaldehyde (PFA) in PBS for 10 min, followed by one 5-min wash with PBS and one 5-min wash with PBST (0.1% Triton X-100 in PBS). Cells were incubated with blocking solution [1% bovine serum albumin (BSA) and 5% normal donkey serum in PBST] for 20 to 30 min, followed by an overnight incubation at 4°C with primary antibodies in blocking solution. Three 5-min PBST washes were performed, followed by a 1-hour incubation with secondary antibodies and DAPI (1 μg/ml) in blocking solution. Two 5-min PBST washes were performed, and the cells were stored in the dark at 4°C in PBST before imaging on a Zeiss LSM980 confocal microscope using a LD C-Apochromat 40×/1.1 water immersion objective. A list of antibodies used and their dilutions is provided in table S4.

Meiotic chromosome spreads were performed as follows: Hypotonic extraction buffer (50 mM sucrose, 17 mM trisodium citrate, 30 mM tris, and 5 mM disodium EDTA, in nuclease-free water, adjusted to pH 8.3 with HCl) was placed on ice and supplemented with 0.5 mM dithiothreitol and 0.5 mM phenylmethylsulfonyl fluoride. Cells from one well of a 12-well plate were harvested with Accutase, spun down (300*g*, 5 min), resuspended in hypotonic extraction buffer (1 ml), and incubated at room temperature for 30 min. Microscope slides were prepared by drawing approximately 2 cm–by–2 cm squares with a hydrophobic pen and placing 100 μl of freshly prepared fixation solution (1% PFA, 0.15% Triton X-100, 0.5 mM boric acid, and 0.5 mM NaOH, in nuclease-free water) into each square. Cells were spun down (100*g*, 3 min) and resuspended in 0.35 ml of 100 mM sucrose in nuclease-free water. Ten microliters of cell suspension was added to each square of the slides, and mixing was performed by gentle swirling. Slides were incubated in a humid chamber for 2 hours at room temperature and then air dried for 3 to 5 hours. Slides were washed three times with 0.4% PhotoFlo (Kodak) in nuclease-free water, air dried for 30 min, and stored at −80°C. Staining of the slides was performed as described above, starting with the blocking solution step. After staining, slides were mounted in ProLong Glass (Invitrogen) and imaged on a Zeiss LSM980 confocal microscope using a LD C-Apochromat 63×/1.4 oil immersion objective.

Initial image processing was performed using FIJI (ImageJ version 2.14.0/1.54f) (*52*). Brightness and contrast were adjusted equally across all images from each experiment. Segmentation and quantification were performed using the cellpose Python package (version 2.2.3) (*53*). Thresholding was performed by using a negative control (typically hiPSCs) to determine the average background staining intensity, subtracting this average background intensity from all regions, and then classifying any region that was >2× brighter than then 95th percentile of the negative control as positive. The analysis code is available on GitHub: https://github.com/mpiersonsmela/meiosis.

For classification of stages of meiosis, segmentation was performed using cellpose on the DAPI channel, and initial thresholding was performed as described above. Nuclei intersecting the edges of the image were excluded. Nuclei that did not exceed the negative control threshold for any channel were classified as nonmeiotic. For each meiotic nucleus, a square region measuring 2× the average nuclear diameter was extracted as a .png file. We manually examined the files (1707 files total) and classified each as leptotene, zygotene, or pachytene-like, or aberrant.

#### Barcode enrichment screening

Reporter iPSCs containing integrated expression transposon vectors were harvested with Accutase. Cells were seeded on Matrigel-coated six-well plates (500,000 cells per plate) in mTeSR1 medium (STEMCELL Technologies) with doxycycline (1 μg/ml) and Y-27632 (10 μM). A media change was performed after 24 hours. At this point, candidate differentiation media were tested, based on previous claims of meiosis induction in the literature. These included

Alpha minimal essential medium (Thermo Fisher Scientific) (Gibco with 1× GlutaMAX, 1× insulin-transferrin-selenium-X supplement, 0.2% BSA, 0.2% chemically defined lipid concentrate, ascorbic acid (200 μg/ml), fibroblast growth factor 2 (FGF2; 1 ng/ml), and glial cell line–derived neurotrophic factor (GDNF; 20 ng/ml) (*54*).

Nutrient restriction/retinoic acid medium is composed of EBSS with 0.1× Iscove’s modified Dulbecco’s medium (IMDM), 0.1× supplements (N2, GlutaMAX, sodium pyruvate, MEM (minimum essential medium) essential vitamins, nonessential amino acids), 0.1% fetal bovine serum, 0.5% knockout serum replacement, glucose (0.6 mg/ml), lactic acid (0.1 mg/ml), BSA (0.5 mg/ml), 5 μM 2-mercaptoethanol, 10 μM ascorbic acid, biotin (1 μg/ml), β-estradiol (3 ng/ml), FGF2 (1 ng/ml), GDNF (1.5 ng/ml), and 100 nM retinoic acid (*9*).

We additionally tested mTeSR1 (which maintains primed pluripotency), Human Enhanced Naïve Stem cell Medium (*55*), StemPro-34–based spermatogonial stem cell culture medium (*56*), and APEL2 (STEMCELL Technologies; a “neutral” medium lacking growth factors).

The cells were cultured in these six media, all supplemented with doxycycline (1 μg/ml), for six additional days. A media change was performed each day. Cells were harvested with Accutase on day 7 postinduction, and reporter-positive cells were isolated by fluorescence-activated cell sorting (FACS). DNA was extracted from REC8^+^, SYCP3^+^, and DDX4^+^ cells, as well as the presorting population and predifferentiation population. Barcodes were amplified by two rounds of PCR and sequenced on an Illumina MiSeq as previously described (*42*). Barcode enrichment was calculated by comparing the barcode frequencies in reporter-positive and presorting cells. We also sequenced barcodes from the predifferentiation cells but did not use these for analysis since effects were dominated by changes in the cell growth rate.

#### CRISPRi of epigenetic factors

Guide RNAs targeting the promoters of 10 epigenetic factors (*MAX*, *MGA*, *E2F6*, *RNF2*, *PCGF6*, *SETDB1*, *DNMT1*, *DNMT3A*, *DNMT3B*, and *UHRF1*; three guides per gene) were cloned into a doxycycline-inducible dCas9-KRAB expression transposon plasmid (*47*). Each transposon plasmid was integrated into SYCP3 reporter iPSCs as described above. iPSCs were treated with doxycycline (1 μg/ml) in mTeSR1 for 6 days, harvested with Accutase, and analyzed by flow cytometry (fig. S4). In addition, knockdown efficiency was evaluated by quantitative PCR (qPCR) with PowerUp SYBR Green Master Mix (Thermo Fisher Scientific) using *GAPDH* as a control gene (fig. S4C). Primers used are given in table S3.

#### Screening of conditions for reporter activation

Several flow cytometry experiments were performed to optimize conditions for activating REC8 and SYCP3 expression in male (PGP1) and female (F3) reporter lines. First, expression vectors for 16 promising factors (fig. S3) chosen on the basis of barcode enrichment data were individually integrated into REC8 and SYCP3 reporter iPSC lines, and expression was induced by treatment with doxycycline (1 μg/ml) in APEL2 medium following the protocol described above in the barcode enrichment section. Second, a fractional factorial screen for REC8 activation was conducted in F3 and PGP1 D4TR8G reporter lines, using 32 combinations of seven promising factors and following the same differentiation protocol (Fig. 1C).

Third, REC8, SYCP3, and DDX4 activation was measured after performing differentiation using expression of STRA8, HOXB5, and BCL2 in the presence of different basal media and additives. Four basal media were tested: mTeSR1 (STEMCELL Technologies), APEL2 ((STEMCELL Technologies), DMEM (Dulbecco’s modified Eagle’s medium)/F12 with 1× GlutaMAX and 10% knockout serum replacement (Gibco), and aRB27 [advanced RPMI with 1× nonessential amino acids, 1× GlutaMAX, and 0.5× B27 supplement minus vitamin A (all from Gibco)]. For each of the differentiation media, five additives were tested, as well as a doxycycline-only control. The additives and their concentrations were 1 μM retinoic acid, 1 μM AM580, 25 μM PRT4165, 20 μM RB3, and 1 mM sodium valproate. A media change was performed every day. At days 6, 7, and 8 postinduction, cells were harvested with Accutase and analyzed by flow cytometry (Fig. 1D)

Fourth, REC8, SYCP3, and DDX4 activation was measured in cells treated with or without DNMT1 inhibitor (5 μM GSK3484862). Differentiation was performed using expression of STRA8, HOXB5, and BCL2 in APEL2 medium supplemented with 1 μM AM580 and doxycycline (1 μg/ml). Cells were harvested with Accutase after 7 days and analyzed by flow cytometry (Fig. 1E).

#### scRNA-seq screening

Before scRNA-seq, the following six cell populations were generated by integration of transposon expression vectors:

1) PGP1 D4TR8G, with BCL2, HOXB5, and STRA8 under hygromycin selection

2) F3 D4TS3G, with BCL2, HOXB5, and STRA8 under hygromycin selection

3) PGP1 D4TR8G, with BCL2, HOXB5, and STRA8 under hygromycin selection and the full pool of 88 candidate factors under puromycin selection

4) F3 D4TS3G, with BCL2, HOXB5, and STRA8 under hygromycin selection and the full pool of 88 candidate factors under puromycin selection

5) PGP1 D4TR8G, with the full pool of 88 candidate factors under puromycin selection

6) F3 D4TS3G, with the full pool of 88 candidate factors under puromycin selection

The cells were differentiated according to the following method, which had been chosen on the basis of its ability to activate REC8 and SYCP3 expression. Cells containing integrated expression vectors were seeded in mTeSR1 containing 10 μM Y-27632, 5 μM GSK3484862, and doxycycline (1 μg/ml). After 24 hours, the medium was changed to APEL2 with 5 μM GSK3484862, 1 μM AM580, and doxycycline (1 μg/ml). A media change was performed every other day. After a total of 7 days of differentiation, cells were harvested with Accutase and sorted on the basis of reporter expression. Cells were fixed using a Parse fixation kit, and scRNA-seq library preparation was performed using a Parse WT Mega v2 kit. A list and description of samples are provided in table S5. Sequencing was performed on an Illumina NovaSeq X Plus using three full 10B PE150 flow cells. Alignment and count matrix generation were performed using the Parse Biosciences pipeline (v.0.9.6).

To enrich the library for barcode sequences, we first performed PCR with biotinylated primers to generate a double-stranded DNA biotinylated bait containing the 120 bp of sequence immediately 3′ of the barcode sequence in our expression vector. We isolated the bait DNA from the PCR using a 3× volume of ProNex beads and eluted in 10 mM tris (pH 8.0) buffer. Next, we used 200 fmol of bait DNA as a custom probe in the Parse Gene Capture kit, following the manufacturer’s protocol aside from this substitution. After qPCR to verify the barcode enrichment, the resulting library was sequenced on one lane of a NovaSeq X Plus 10B PE150 flow cell.

scRNA-seq data were filtered by the number of reads per cell (<100,000), number of genes detected (>1000 and <14,000), and mitochondrial read percentage (<10%). Transgene barcode reads were merged into the dataset by matching cell barcodes. Analysis was performed using scanpy (*57*) for normalization, integration with the fetal germ cell reference atlas (*20*), and gene scoring

#### Refinement of factors for meiosis induction

Several experiments were performed to optimize the protocol for meiosis induction. First, for each of the 23 candidate factors identified by the scRNA-seq screen (excluding HOXB5, which was already integrated), an expression vector with a puromycin selection marker was integrated into PGP1 D4TR8G and F3 D4TS3G reporter hiPSCs which already contained expression vectors for BCL2, HOXB5, and STRA8 with hygromycin selection markers. Two control conditions were additionally included: BCL2, HOXB5, and STRA8 only; and a no-factor control. Cells were differentiated using the same conditions as for the scRNA-seq experiment (APEL2 with doxycycline, AM580, and GSK3484862). Cells were fixed and stained for SYCP3, HORMAD1, and DDX4 after 7 days of differentiation.

Second, expression vectors for the seven top factors identified in the previous experiment (BCL2, HOXB5, STRA8, myr-AKT1, BOLL, MEIOC, and MEIOSIN) were pooled and integrated into PGP1 D4TR8G and F2 D4TDZG hiPSCs. Cells were differentiated in the same manner, and fixation and staining for SYCP3, HORMAD1, and γH2AX were performed after 7, 9, 13, and 16 days of differentiation. As a control, the same differentiation and staining were performed using only BCL2, HOXB5, and STRA8.

Third, a fractional factorial screen was performed to identify the contributions of the seven top factors. Sixteen combinations of the seven factors (including one control combination lacking all factors) were integrated into F2 D4TDZG, F3 D4TS3G, and PGP1 D4TR8G reporter lines. In addition, expression vectors for DAZL and BOLL were integrated to evaluate previous claims that those factors alone could induce meiosis (*14*, *17*). Cells were fixed and stained for SYCP3, HORMAD1, and γH2AX after 13 days of differentiation. Additional cells were analyzed by flow cytometry for reporter expression.

Fourth, a full factorial screen was performed to confirm the best factors for meiosis induction. A constitutive EF1a-driven BCL2 expression plasmid was integrated into F2 D4TDZG, F3 D4TT2G, and PGP1 D4TR8G reporter hiPSCs under hygromycin selection. Then, all eight possible combinations of HOXB5, BOLL, and MEIOC expression vectors were integrated under puromycin selection. Cells were fixed and stained for after 13 days of differentiation, with the first 3 days at 37°C and the remainder at 34°C. Two stains were used: SYCP3, HORMAD1, and γH2AX; and TEX12 and SYCP3.

#### Timing of media additives and factor expression

A Shield1-inducible HOXB5 PiggyBac transposon plasmid was constructed using an EF1a promoter driving HOXB5 with a C-terminal degradation domain, which could be stabilized by addition of Shield1. This plasmid, along with expression plasmids for constitutive EF1a-driven BCL2 and doxycycline-inducible BOLL, was integrated into F2 D4TDZG, F3 D4TS3G, and PGP1 D4TR8G reporter lines. Cells were seeded at a density of 50,000/cm^2^ in Matrigel-coated 96-well ibidi plates in mTESR1 + 10 μM Y-27632. After 24 hours, the medium was changed to APEL2. Subsequently, a full media change was performed every 48 hours, and cells were fixed and stained for HORMAD1, SYCP3, and γH2AX after 11 days of differentiation. This experiment was performed at 37°C. A complete list of conditions tested and corresponding outcomes is listed in table S6 and shown in fig. S6.

#### Evaluation of temperatures and timings for meiosis induction

As an initial experiment to evaluate the effects of lower temperature on male meiosis, PGP1 D4TR8G reporter hiPSCs containing integrated expression vectors for BCL2, HOXB5, BOLL, and MEIOC were seeded in eight-well ibidi dishes at 50,000 cells/cm^2^ in mTeSR1 with 5 μM GSK3484862, doxycycline (1 μg/ml), and 10 μM Y-27632. After 24 hours, the media was replaced with APEL2 containing 5 μM GSK3484862, 1 μM AM580, and doxycycline (1 μg/ml). A 50% media change was performed every 2 days, and GSK3484862 was withdrawn starting on day 7. Initially, all cells were cultured at 37°C. One plate was moved to a 34°C incubator after the first day. Cells were fixed on day 13, stained for HORMAD1, SYCP3, and γH2AX, and imaged.

As a confirmatory experiment, F2 D4TDZG, F3 D4TS3G, and PGP1 D4TR8G reporter hiPSCs containing integrated expression vectors for BCL2, HOXB5, BOLL, and MEIOC were differentiated according to the same protocol. The following conditions were tested:

1) 34°C starting on day 3, fixation at day 13

2) 34°C starting on day 1, fixation at day 14

3) 34°C starting on day 3, fixation at day 15

4) 34°C starting on day 3, fixation at day 16

5) Continuous 37°C, fixation at day 16

6) 34°C starting on day 1, fixation at day 17

7) 34°C starting on day 3, fixation at day 19

8) 34°C starting on day 3, fixation at day 21

After fixation, cells were stained for HORMAD1, SYCP3, γH2AX, and actin and imaged.

#### Optimized protocol for meiosis induction

On the basis of the results of our screening and optimization experiments, which are described above, we have developed the following protocol for robust initiation of meiosis: A constitutive or doxycycline-inducible expression vector for the antiapoptotic factor BCL2, as well as doxycycline-inducible expression vectors for meiosis-promoting factors (HOXB5, BOLL, and/or MEIOC), is integrated into human iPSCs using PiggyBac transposase. The iPSCs are seeded at 50,000 cells/cm^2^ on Matrigel-coated or laminin-511–coated plates in mTeSR1 supplemented with 5 μM GSK3484862, doxycycline (1 μg/ml), and 10 μM Y-27632 and incubated at 37°C. For six-well plates, 1.5 ml of medium is used per well, and volumes for smaller plates are scaled down proportionally to their surface area. One day after seeding the cells, the media is replaced with APEL2 containing 5 μM GSK3484862, 1 μM AM580, and doxycycline (1 μg/ml). A 50% media change is performed every 2 days. Three days after seeding the cells, they are moved to a 34°C incubator. GSK3484862 is withdrawn starting on day 7. Initiation of meiosis is complete by roughly day 13.

#### Time course scRNA-seq and imaging

Plasmids for constitutive expression of BCL2 and doxycycline-inducible expression of HOXB5, BOLL, and MEIOC were integrated into F2 D4TDZG, F3 D4TT2G, and PGP1 D4TR8G reporter hiPSCs. Meiosis was initiated following the final optimized protocol, with cells cultured on 8-well ibidi dishes for immunofluorescence imaging and 12-well plates for scRNA-seq. At each day from day 0 (hiPSC) to day 15, cells were fixed for imaging and harvested for scRNA-seq. Stains used for imaging were as follows: rabbit anti-HORMAD1, goat anti-SYCP3, mouse anti γH2AX, and rat anti-T2A; and rabbit anti-TEX12, goat anti-SYCP3, mouse anti-RAD51, and rat anti-KI67. Samples for scRNA-seq were counted and fixed using the Parse Biosciences fixation kit. Library preparation was performed using the Parse Biosciences WT v3 kit. Sequencing was performed on two lanes of a NovaSeq X Plus 25B PE150 flow cell. Alignment and counts matrix generation were performed using the Parse Biosciences pipeline (v.1.2.1). Filtering, normalization, and cell type annotation were performed in scanpy as described above. To build a reference atlas, we combined the fetal gonad atlas and adult testis atlas (*20*, *23*), removing somatic cell types from the testis atlas and only keeping genes that were expressed in both atlases.

#### Methyl-seq

In a pilot experiment, DNA was extracted from fixed cells from the time course scRNA-seq experiment (days 0, 1, 2, 3, 4, 5, 6, 7, 9, 11, and 13 and 15 time points; three cell lines per time point; total of 36 samples) using the QIAamp DNA Micro FFPE kit (QIAGEN). Two hundred nanograms of DNA per sample was sheared to an average of 300 bp using a Covaris S2, and libraries were prepared using the New England Biolabs Enzymatic Methyl-Seq kit following the manufacturer’s protocol. Libraries were sequenced on an Illumina MiSeq and processed using the Nextflow Methylseq pipeline (v. 2.6.0). In the follow-up experiment, DNA was extracted using the QIAamp DNA Micro kit (QIAGEN) from cells at day 0 (hiPSC) and day 2 during the meiosis induction. At days 5, 10, and 15, DDX4^+^ and DDX4^−^ cells were isolated by FACS for the DDX4-T2A-tdTomato reporter, and DNA was similarly extracted. Three replicate hiPSC lines were used for each condition (total 24 samples). Libraries were prepared as described above and sequenced on two lanes of a NovaSeq X Plus 25B flow cell (yielding 11× to 14× genome-wide coverage per sample). Initial processing was performed using the Nextflow Methylseq pipeline (v. 2.6.0). Methylation analysis of genomic features was performed using CpGtools (*58*) with genomic feature files obtained from the UCSC hg38 database (*59*) or the Human Imprintome (*60*). For promoter analysis, the promoter region was defined as 1000 to the 3′ direction and 100 bp to the 5′ direction around each transcriptional start site. Differentially methylated regions were calculated using Dispersion Shrinkage for Sequencing (DSS) (*61*).

#### Single-cell multiomics

Cryopreserved cells from day 15 of the time course scRNA-seq experiment (from F2 D4TDZG, F3 D4TT2G, and PGP1 D4TR8G reporter lines) were processed for 10× Multiome ATAC + 3′ RNA-seq library prep by Admera Health. Sequencing (150-bp paired end reads) of RNA and ATAC libraries was performed on an Illumina NovaSeq X Plus. Across three samples, we obtained data from 36,534 cells at an average read depth of 16,774 (RNA) and 25,088 (ATAC) reads per cell.

The Cell Ranger ARC pipeline (10x Genomics, version 2.0.2) was used for alignment, data processing, and calculation of quality control metrics including transcriptional start site enrichment scores. Further processing was performed using a code available at https://github.com/mpiersonsmela/meiosis. In summary, RNA expression was analyzed in scanpy (version 1.10.3), and cell type annotation was performed using scanpy ingest with the fetal gonad 10× scRNA-seq reference atlas (E-MTAB-10551) (*20*). The SCENIC+ pipeline (*25*) was used to identify consensus ATAC peaks. We then intersected these consensus peaks with genomic features of interest (as described for the methyl-seq analysis). Overlaps were quantified as the proportion of consensus peak bases intersecting each feature category,

To determine how closely our meiotic cells resemble atlas germ cells at the chromatin level, we performed a parallel joint clustering of RNA-seq and ATAC-seq data from our cells and the fetal gonad reference multiome dataset (E-MTAB-11708). To compare two sets of different regions (each obtained via the SCENIC+ ATAC preprocessing step, once for our in vitro meiotic cells and once for the reference atlas), we merged them using pybedtools merge. Each fragment matrix was then projected onto the new region set by length-of-overlap weights. To correct for batch effects across the different datasets, we used scanpy ingest on the fragment matrices and then replotted the joint clustering.

DARs between meiotic oogonia and their in vivo counterparts were identified using DESeq2 (version 1.42.1). We used a pseudobulk approach, summing up the fragment matrices at the cell type, sample, and dataset levels. Annotation of DARs was performed using the algorithm in 10X CellRanger ARC, with the Gencode basic gene annotation feature file (GRCh38.p14, release 47). GO term enrichment analysis was performed using the PantherDB application programming interface (*62*), setting a cutoff of abs(log_2_ fold change) > 3 and *P* < 0.05. To compute the Spearman correlation coefficients on average gene expression and quantify the extent of similarity among corresponding cell types, we used a similar pseudobulk approach, summing up normalized gene expressions at the dataset and cell type levels.

Last, we ran the SCENIC+ pipeline using the prebuilt AertsLab cisTarget human ranking and score databases. We defined meiotic cells as oogonia_meiotic and oogonia_STRA8 cell types. We used the difference between the regulon specificity scores of meiotic cells and nonmeiotic cells as a scoring function for enriched eRegulons in meiotic cells. We also aggregated the results at the TF level to better identify potential TFs responsible for meiosis.

#### Live-cell imaging

For live-cell imaging, cells expressing mStayGold and tdTomato reporters were grown in ibidi eight-well dishes, stained with Hoechst 33342 (5 μg/ml in PBS) for 15 min at 34°C, then washed twice with PBS, and imaged on the Zeiss LSM980 confocal microscope using the 40× water objective.

### Statistical analysis

Results of fractional factorial screens were analyzed by fitting linear models using the lm function in R (version 4.3.2). For flow cytometry data, values expressed as a proportion (0 to 100%) were logit-transformed before fitting the model. Significance calculations in bar plots were performed using two-tailed Mann-Whitney *U* tests. Error bars represent 95% confidence intervals. Significance calculations for promoter methylation were performed using paired two-tailed *t* tests. All sample size values represent the number of distinct biological samples not the repeated measures of the same sample.
