# Supplementary material for: Initiation of meiosis from human iPSCs under defined conditions through identification of regulatory factors
Source: Sci Adv. 2025 Aug 15;11(33):eadu0384. doi: 10.1126/sciadv.adu0384 (PMC12356269; doi:10.1126/sciadv.adu0384)
Supplement: Supplementary file 1 — Figs. S1 to S14 Tables S1 to S7 Legends for tables S8 and S9 Legend for movie S1 [file sciadv.adu0384_sm.pdf]

Science Advances
AAAS

Supplementary Materials for

Initiation of meiosis from human iPSCs under defined conditions through identification of regulatory factors

Merrick Pierson Smela *et al.*

Corresponding author: George M. Church, gchurch@genetics.med.harvard.edu

Sci. Adv. **11**, eadu0384 (2025)
DOI: 10.1126/sciadv.adu0384

The PDF file includes:

Figs. S1 to S14
Tables S1 to S7
Legends for tables S8 and S9
Legend for movie S1

Other Supplementary Material for this manuscript includes the following:

Tables S8 and S9
Movie S1

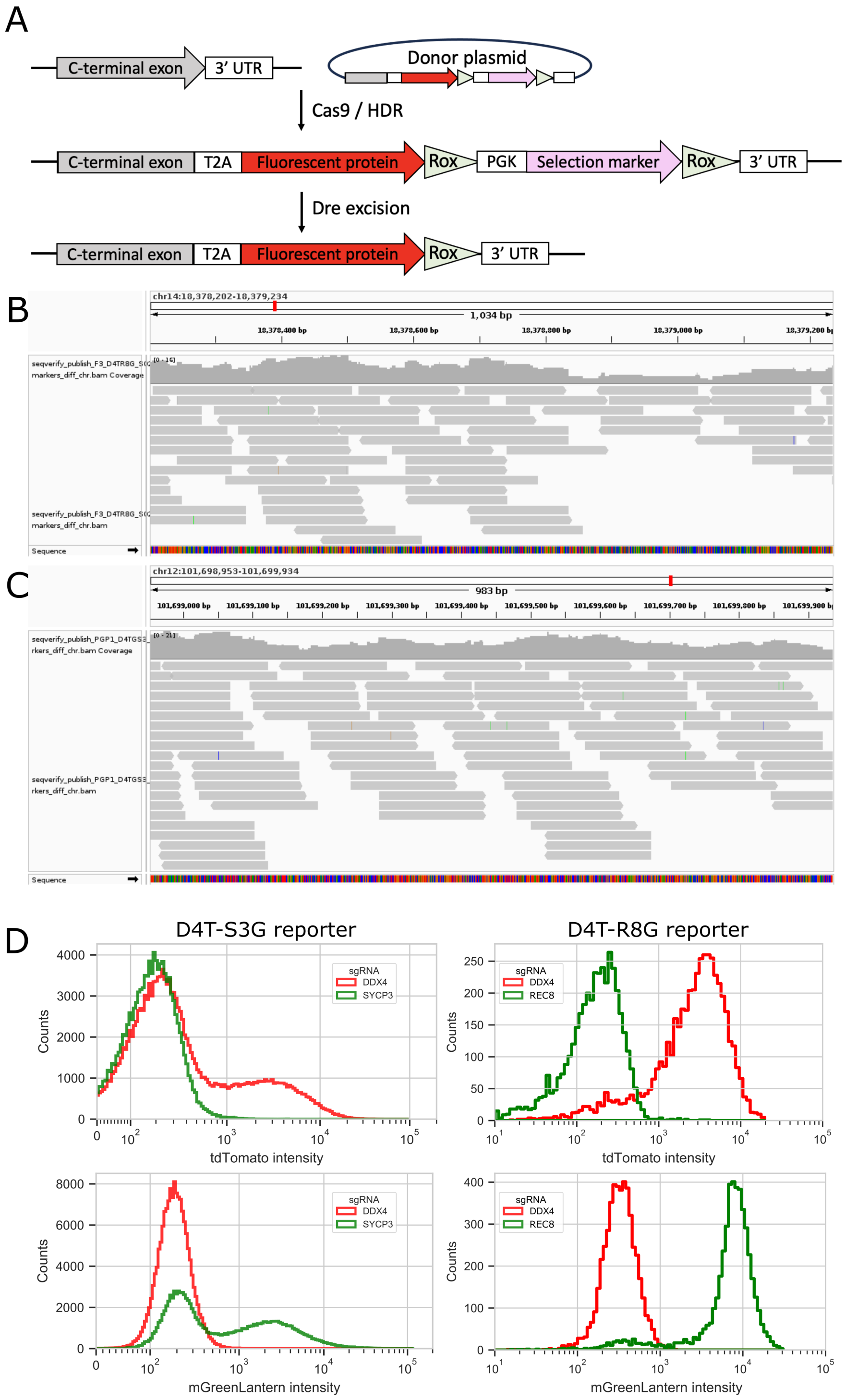

Fig. S1. Construction and validation of hiPSC reporter lines. (**A**) Knock-in editing strategy. (**B**) SeqVerify validation of REC8 reporter allele using whole genome sequencing. (**C**) SeqVerify validation of SYCP3 reporter allele using whole genome sequencing. (**D**) Functional validation of reporter hiPSCs using flow cytometry and CRISPRa with gRNAs targeting the promoters of *REC8, SYCP3*, and *DDX4*.

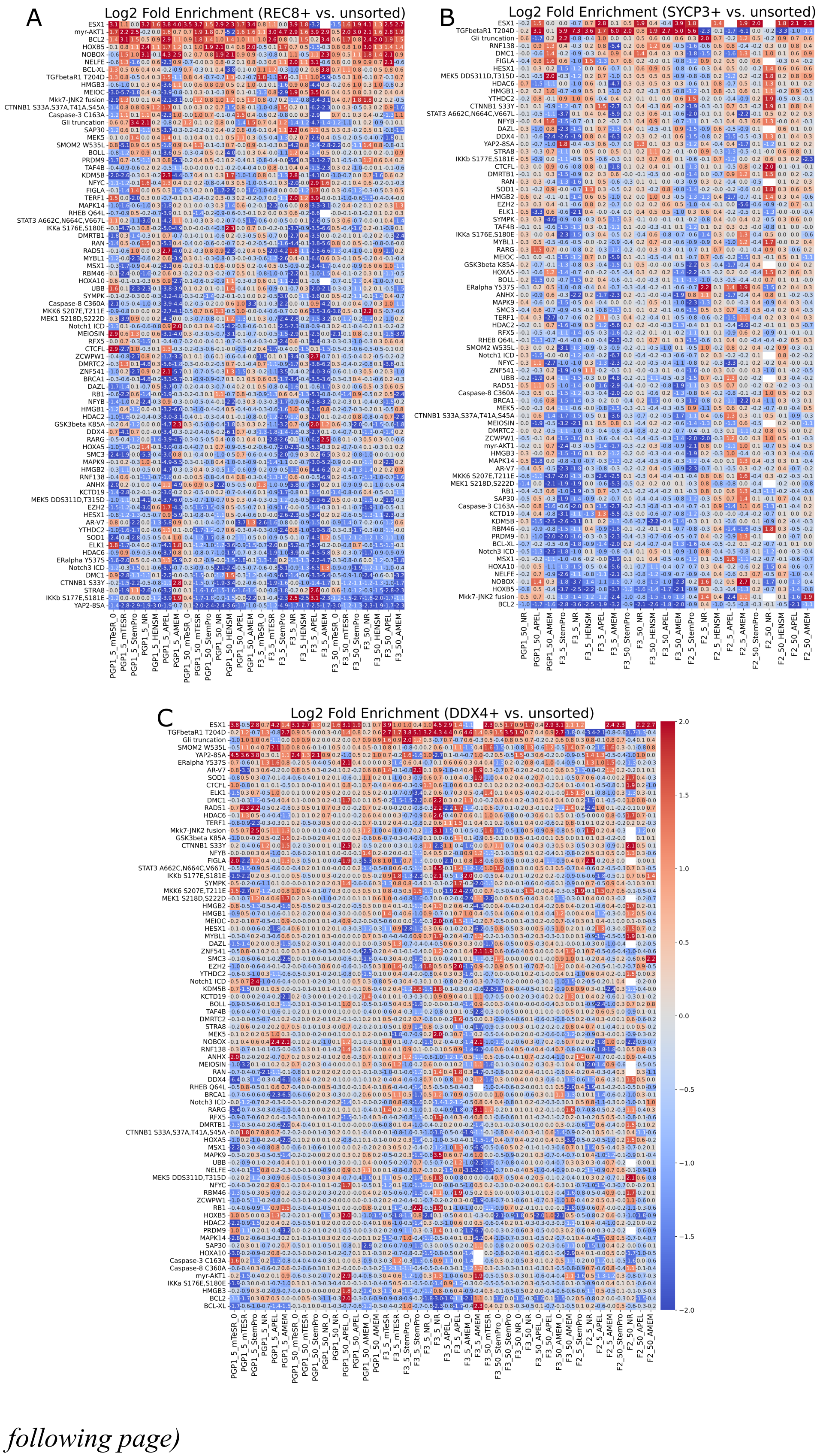

(caption on following page)

Fig. S2. Barcode enrichment results for (**A**) REC8, (**B**) SYCP3, and (**C**) DDX4. Reporter hiPSCs (F2, F3, and PGP1) were nucleofected with low (5 fmol) or high (50 fmol) doses of plasmid library pool, treated with doxycycline to induce expression, and differentiated in various media (mTeSR, StemPro, nutrient restriction (NR), HENSM, spermatogonial stem cell medium (AMEM), and APEL2). Reporter-positive cells were sorted after 7 days, and barcode frequencies were compared to unsorted cells.

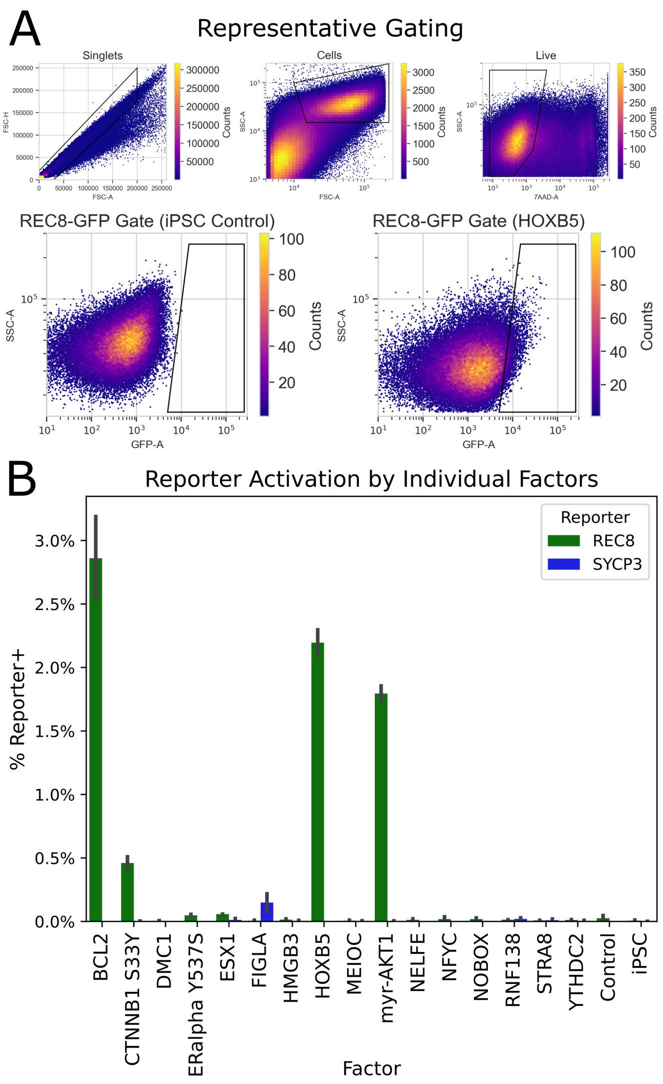

Fig. S3. Flow cytometry analysis of reporter expression induced by individual factors. (**A**) Representative gating strategy for singlets, cells, live cells, and reporter-positive cells. (**B**) Activation of REC8 and SYCP3 reporters by sixteen individual factors chosen based on barcode enrichment results (n = 2 biological replicates per factor per reporter). Error bars represent 95% confidence intervals.

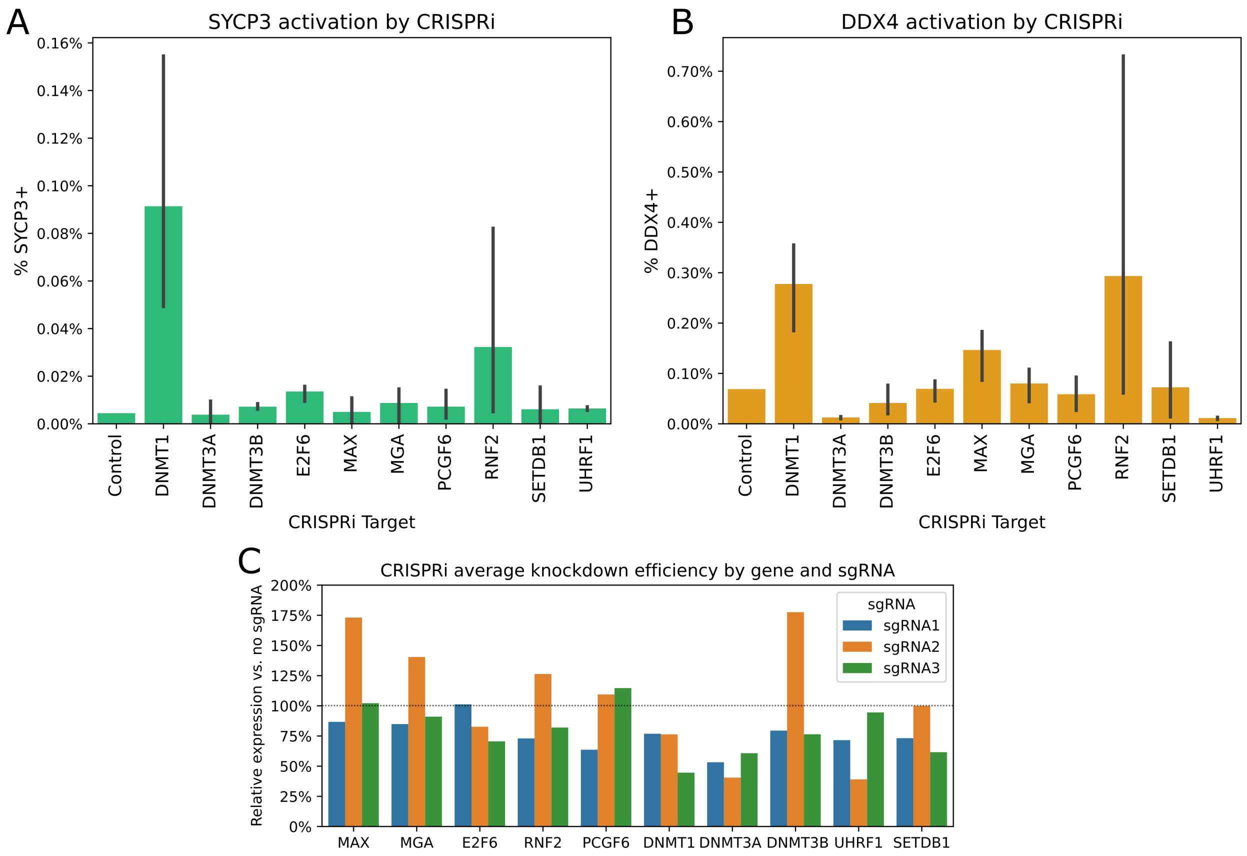

Fig. S4. Pilot screen for activation of SYCP3 and DDX4 expression upon CRISPRi knockdown of ten epigenetic modifiers. (**A**) Activation of SYCP3 expression measured by flow cytometry (n = 3 sgRNAs per gene). (**B**) Activation of DDX4 expression measured by flow cytometry (n = 3 sgRNAs per gene). (**C**) qPCR measurement of average knockdown efficiency (n = 2 technical replicates per guide), calculated by $2^{-\Delta\Delta Ct}$ with *GAPDH* as a reference gene. The bulk knockdown efficiency was poor for most guides, although it is possible that subpopulations of cells experienced a greater knockdown.

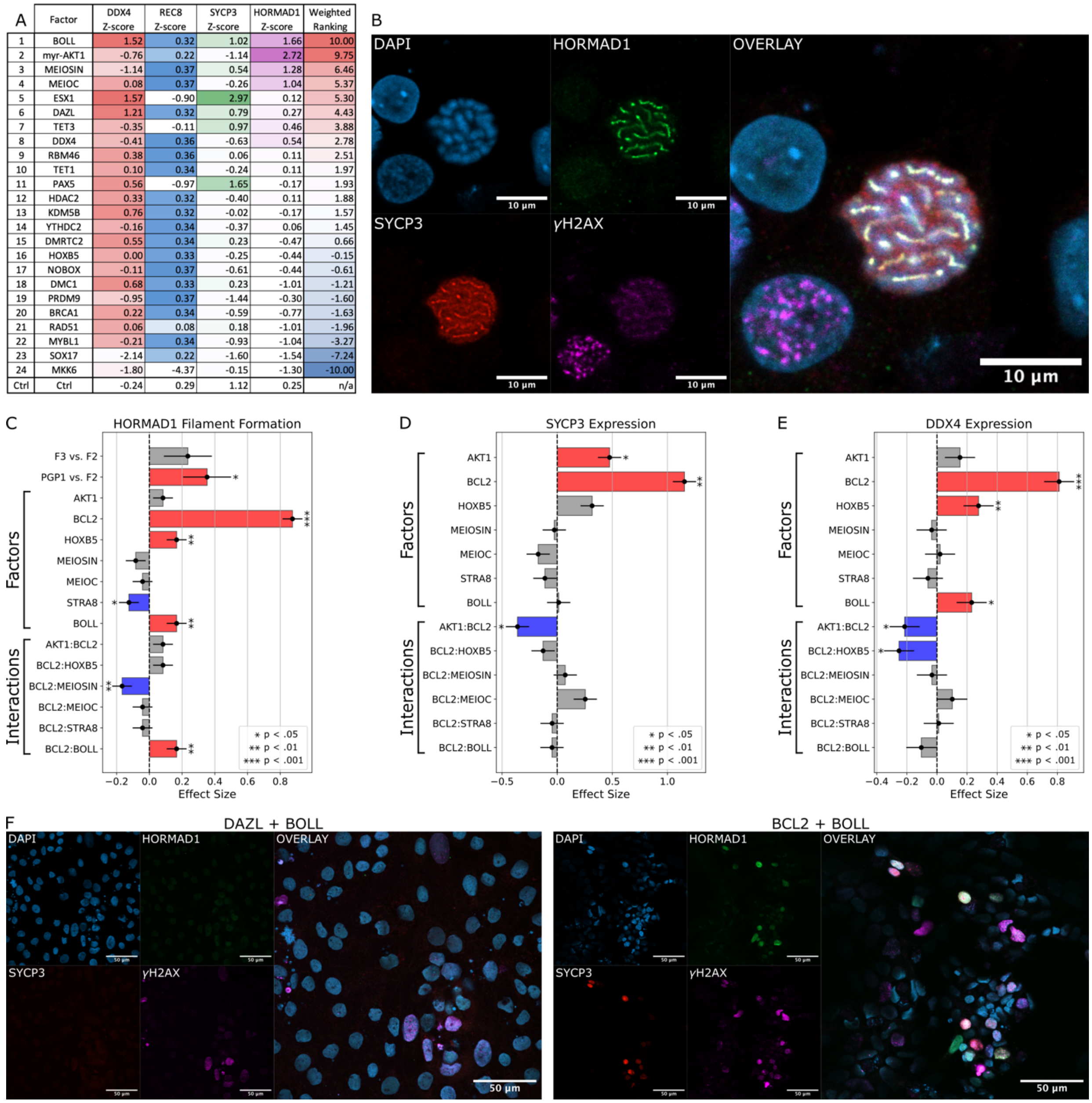

A	Factor	DDX4 Z-score	REC8 Z-score	SYCP3 Z-score	HORMAD1 Z-score	Weighted Ranking
1	BOLL	1.52	0.32	1.02	1.66	10.00
2	myr-AKT1	-0.76	0.22	-1.14	2.72	9.75
3	MEIOSIN	-1.14	0.37	0.54	1.28	6.46
4	MEIOC	0.08	0.37	-0.26	1.04	5.37
5	ESX1	1.57	-0.90	2.97	0.12	5.30
6	DAZL	1.21	0.32	0.79	0.27	4.43
7	TET3	-0.35	-0.11	0.97	0.46	3.88
8	DDX4	-0.41	0.36	-0.63	0.54	2.78
9	RBM46	0.38	0.36	0.06	0.11	2.51
10	TET1	0.10	0.34	-0.24	0.11	1.97
11	PAX5	0.56	-0.97	1.65	-0.17	1.93
12	HDAC2	0.33	0.32	-0.40	0.11	1.88
13	KDM5B	0.76	0.32	-0.02	-0.17	1.57
14	YTHDC2	-0.16	0.34	-0.37	0.06	1.45
15	DMRTC2	0.55	0.34	0.23	-0.47	0.66
16	HOXB5	0.00	0.33	-0.25	-0.44	-0.15
17	NOBOX	-0.11	0.37	-0.61	-0.44	-0.61
18	DMC1	0.68	0.33	0.23	-1.01	-1.21
19	PRDM9	-0.95	0.37	-1.44	-0.30	-1.60
20	BRCA1	0.22	0.34	-0.59	-0.77	-1.63
21	RAD51	0.06	0.08	0.18	-1.01	-1.96
22	MYBL1	-0.21	0.34	-0.93	-1.04	-3.27
23	SOX17	-2.14	0.22	-1.60	-1.54	-7.24
24	MKK6	-1.80	-4.37	-0.15	-1.30	-10.00
Ctrl	Ctrl	-0.24	0.29	1.12	0.25	n/a

Fig. S5. Optimization of factors for meiosis induction. (**A**) 24 candidate factors identified by an scRNAseq screen (Fig. 2D) were each co-expressed with STRA8, BCL2, and HOXB5. Reporter expression was analyzed by flow cytometry, and HORMAD1 expression was analyzed by immunofluorescence microscopy. Factors were ranked according to the results. (**B**) HORMAD1 and SYCP3 filament formation observed by immunofluorescence microscopy after twelve days of expression of seven top factors (STRA8, BCL2, HOXB5, BOLL, AKT1, MEIOSIN, and MEIOC). Scale bars are 10 μm. (**C**) Results of a fractional factorial screen of the seven top factors, for HORMAD1 filament formation. Error bars represent standard errors of linear model coefficient estimates. (**D**) Results for SYCP3 expression. (**E**) Results for DDX4 expression. (**F**) Comparison of DAZL+BOLL overexpression with BCL2+BOLL overexpression. Scale bars are 50 μm.

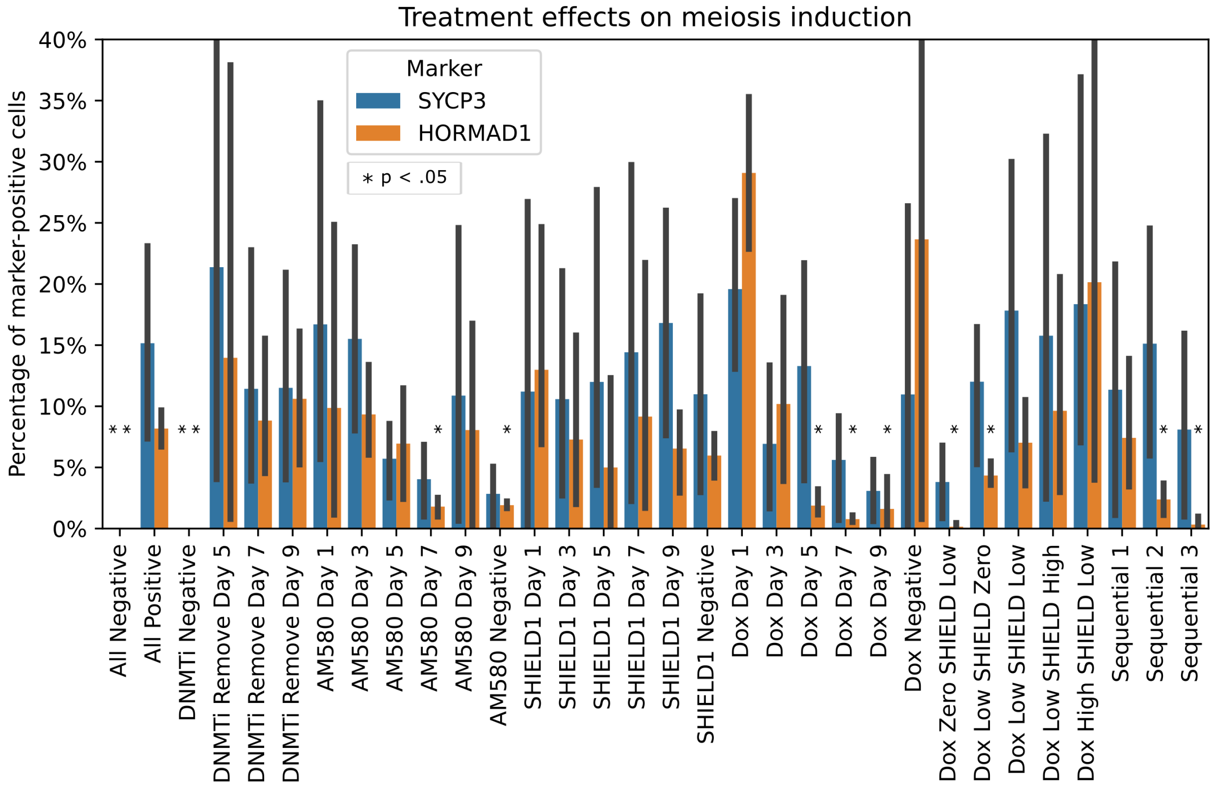

Fig. S6. Quantification of the effects of omitting various components of the meiosis induction protocol, or reducing their doses. Cells constitutively expressed BCL2, and doxycycline was used to activate BOLL expression and Shield1 was used to activate HOXB5 expression over an 11-day meiosis induction. Media supplements (DNMT1i, AM580, doxycycline, and Shield1) were added or omitted as described in Supplementary Table 6. Three biological replicates were tested for each condition. Significance test comparisons (2-tailed *t*-tests) are to the "all positive" control. Error bars represent 95% confidence intervals.

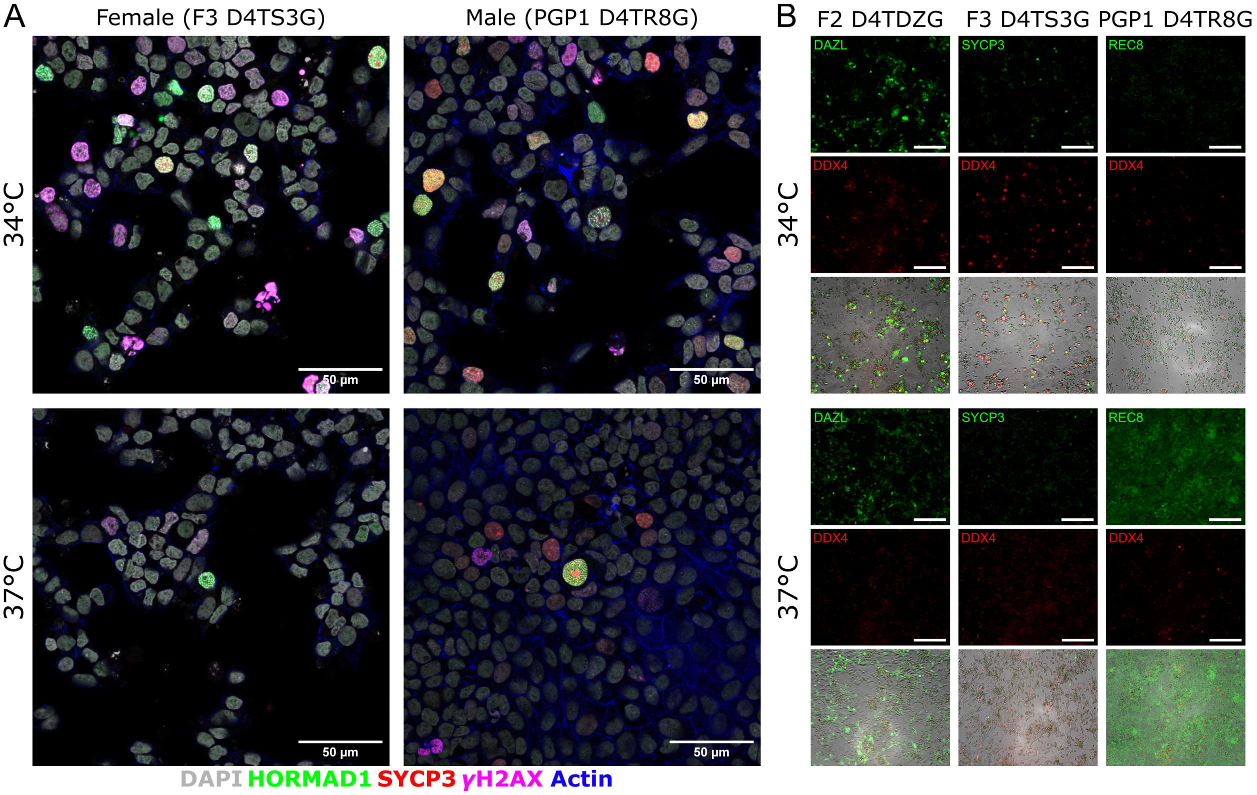

Fig. S7. Microscope images of meiosis induction from male and female hiPSCs at 34 °C vs. 37 °C. (**A**) Immunofluorescence microscopy, staining for DNA (DAPI; gray), HORMAD1 (green), SYCP3 (red), γH2AX (magenta), and actin (phalloidin; blue). Scale bar is 50 µm. (**B**) Live imaging of fluorescent reporter expression of DAZL, SYCP3, REC8, and DDX4. Scale bar is 200 µm.

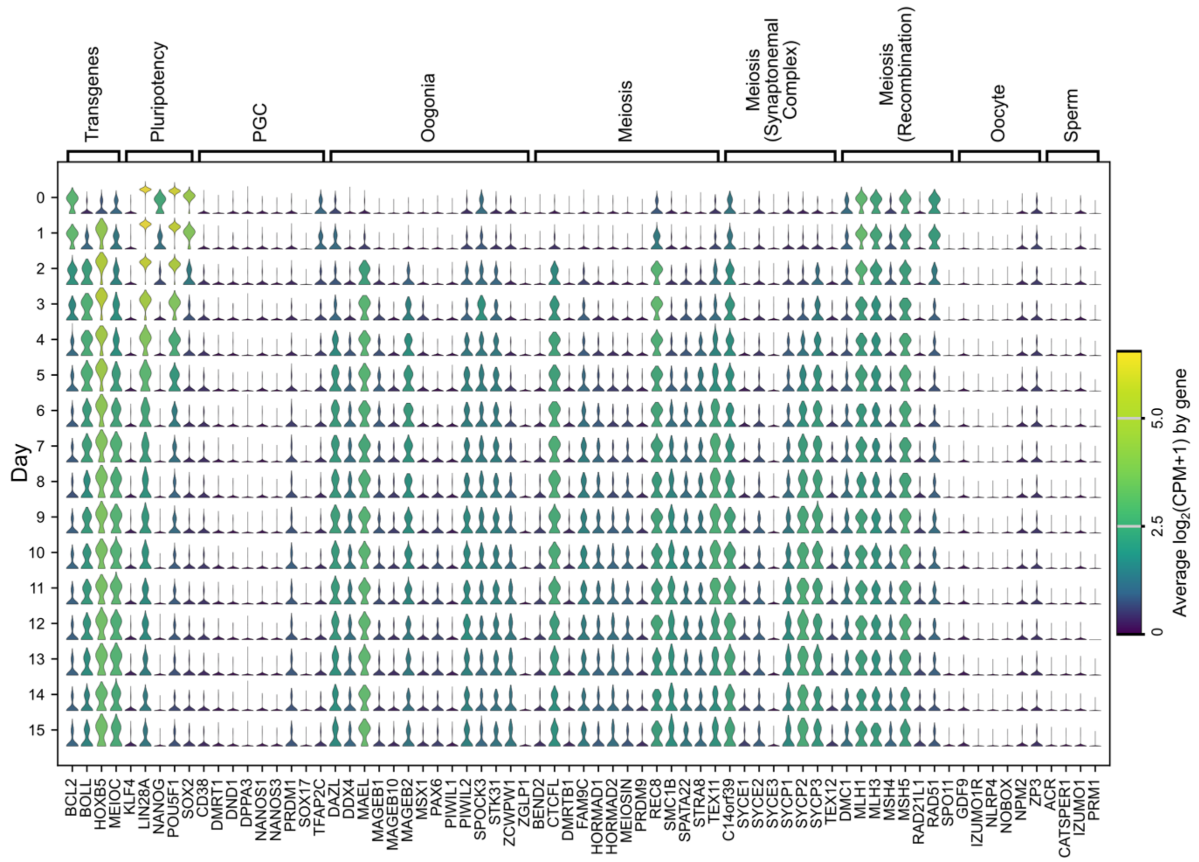

Fig. S8. Violin plots showing expression of marker genes from days 0 to 15 of meiosis induction. Units for the color scale are $\log_2$(CPM+1). Categories include: exogenous transgenes, pluripotency markers, primordial germ cell markers, oogonia markers, meiosis markers, synaptonemal complex components, recombination markers, oocyte markers, and sperm markers.

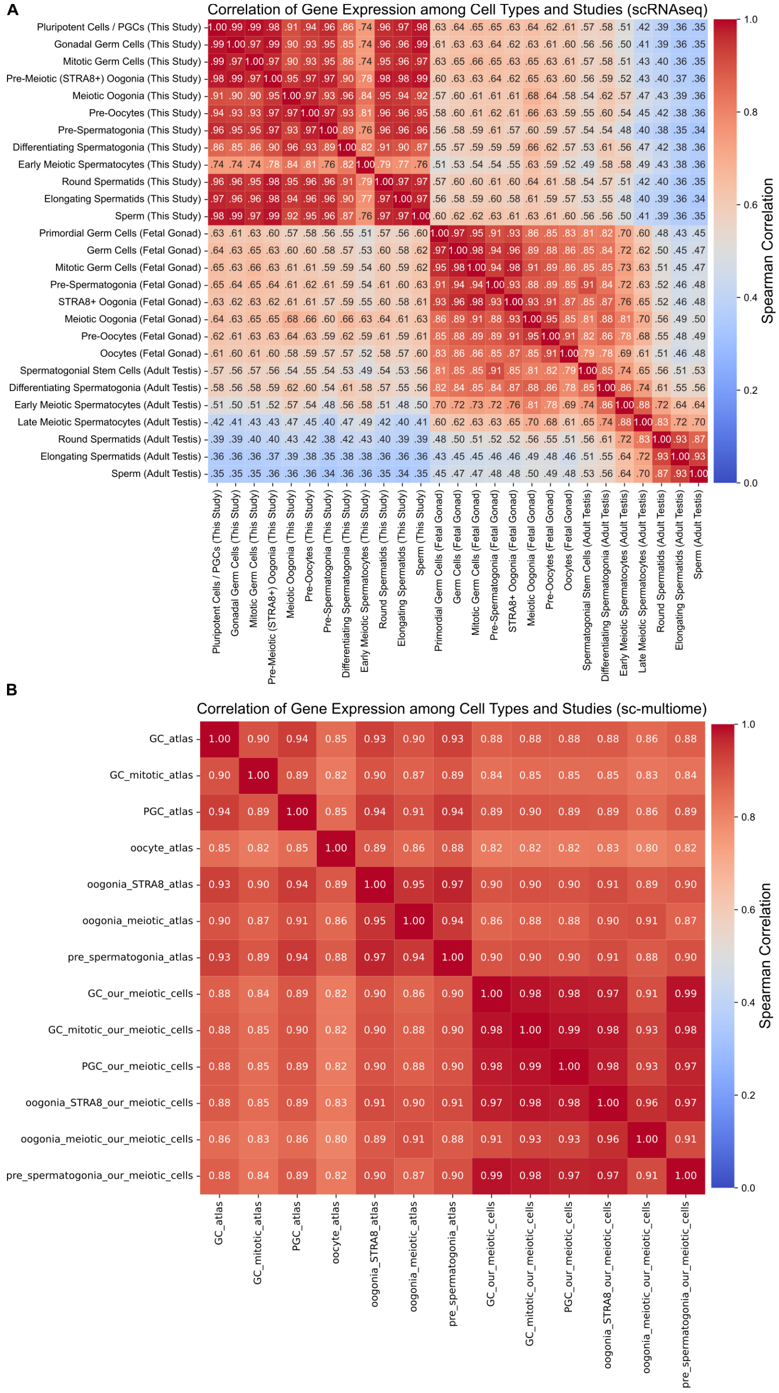

Fig. S9. Gene expression correlation between our cells and corresponding *in vivo* cell types. (A) Spearman correlation matrix of pseudo-bulk gene expression between cell types in this study (15-day meiosis induction; Parse scRNAseq), cell types in the fetal gonad reference atlas (Garcia-Alonso *et al.* 2022; 10X scRNAseq), and cell types in the adult testis reference atlas (Guo *et al.* 2018; 10X scRNAseq), (B) Spearman correlation matrix of pseudo-bulk gene expression between cell types in this study (day 15 of 15-day meiosis induction; 10X multiomics RNA component) and the fetal gonad reference atlas (Garcia-Alonso *et al.* 2022; 10X multiomics RNA component).

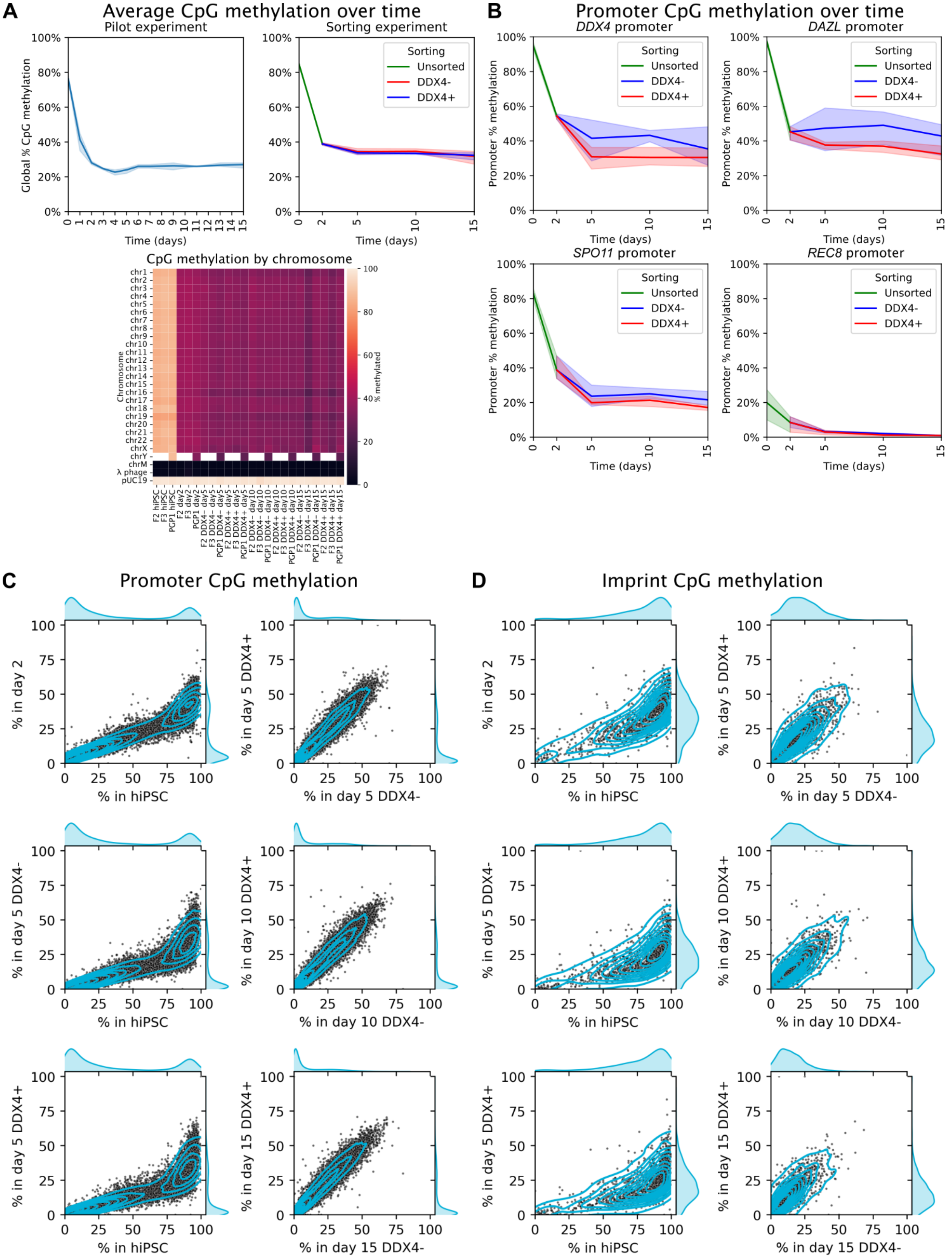
A
Average CpG methylation over time
Pilot experiment
Sorting experiment
Global % CpG methylation
Time (days)
Sorting
Unsorted
DDX4-
DDX4+
CpG methylation by chromosome
Chromosome
% methylated
B
Promoter CpG methylation over time
DDX4 promoter
DAZL promoter
SPO11 promoter
REC8 promoter
Promoter % methylation
C
Promoter CpG methylation
% in day 2
% in hiPSC
% in day 5 DDX4+
% in day 5 DDX4-
% in day 10 DDX4+
% in day 10 DDX4-
% in day 15 DDX4+
% in day 15 DDX4-
D
Imprint CpG methylation

Fig. S10. Analysis of DNA methylation during meiosis induction. (A) Genome-wide methylation for the pilot experiment (12 time points, n = 3 samples per time point) and sorting experiment (8 conditions, n = 3 samples per condition). Unmethylated λ phage and methylated pUC19 spike-in controls show successful library preparation. (B) Promoter methylation of selected oogonia and meiosis marker genes (n = 3 samples per condition). (C) Comparison of promoter methylation between different sample conditions (n = 3 samples per condition). (D) Comparison of imprint control region methylation between different sample conditions (n = 3 samples per condition).

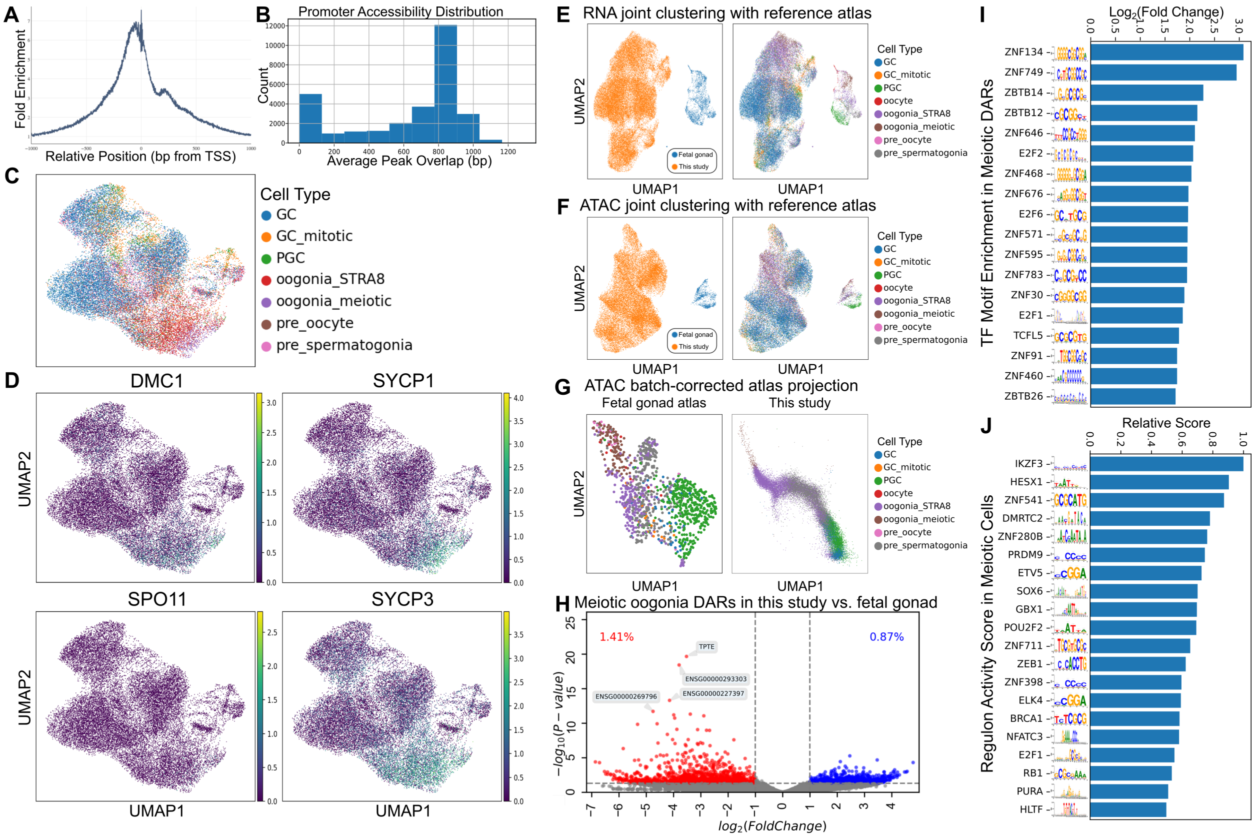

Fig. S11. Multi-omics analysis of cells at day 15 of meiosis induction. (A) Enrichment of ATAC peaks close to transcriptional start sites. (B) Average accessibility of promoters shows a bimodal distribution of open vs. closed states. (C) UMAP plot of RNA expression, with cell types annotated based on projection of RNA expression onto the fetal gonad atlas scRNAseq dataset (*20*). (D) Expression of selected meiotic genes. Color scale is $\log_2(CPM*100 + 1)$. (E) RNA joint clustering of our multi-omics dataset and the fetal gonad atlas multi-omics dataset. (F) ATAC joint clustering of our multi-omics dataset and the fetal gonad atlas multi-omics dataset. Cell types are labeled based on RNA expression. (G) Projection of our multi-omics dataset onto the fetal gonad atlas multi-omics dataset, after batch effect correction with scanpy ingest. Cell types are labeled based on RNA expression. (H) Differentially accessible regions (DARs) between cells annotated as meiotic oogonia in our dataset vs. cells annotated as meiotic oogonia in the fetal gonad atlas multi-omics dataset. The four most significant DARs are labeled. (I) Top 20 TF motifs enriched in DARs between meiotic and non-meiotic cells in our dataset. (J) Top 20 TF regulons specific to meiotic cells (vs. non-meiotic cells) in our dataset, calculated by SCENIC+.

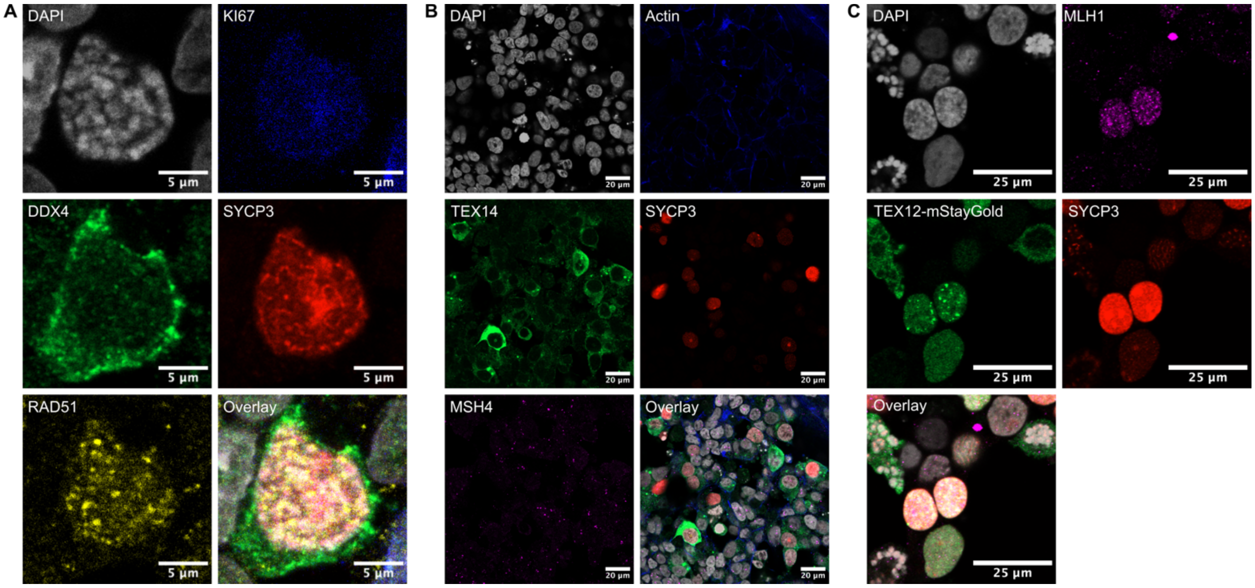

Fig. S12. Immunofluorescence images of marker proteins in meiotic cells. (A) Staining for DNA (DAPI; gray), KI67 (blue), DDX4 (green), SYCP3 (red), and RAD51 (yellow). Scale bar is 5 µm. (B) Staining for DNA (DAPI; gray), actin (phalloidin; blue), TEX14 (green), SYCP3 (red), and MSH4 (magenta). Scale bar is 20 µm. (**C**) (A) Staining for DNA (DAPI; gray), MLH1 (magenta) and SYCP3 (red), with additional imaging of TEX12-mStayGold (green). Scale bar is 25 µm.

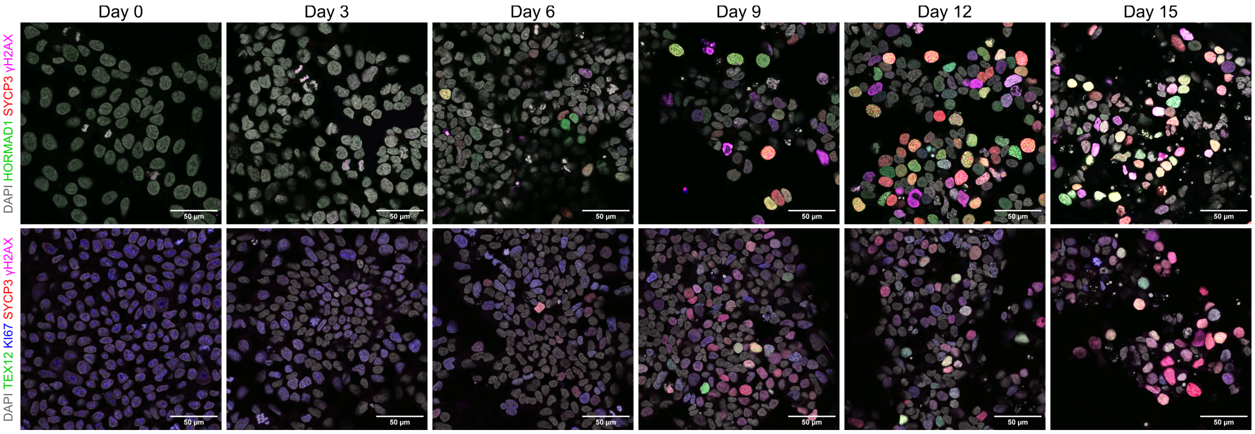

Fig. S13. Representative immunofluorescence images of meiotic marker protein expression over a 15-day timecourse. iPSCs were induced to initiate meiosis using the optimized protocol with constitutive expression of BCL2 and Dox-inducible expression of HOXB5, BOLL, and MEIOC. Cells were fixed on the days indicated and stained for the following markers: SYCP3 (red), HORMAD1 (green), and γH2AX (magenta) (top row); SYCP3 (red), TEX12 (green), KI67 (blue), and γH2AX (magenta) (bottom row). All cells were additionally stained with DAPI (gray). Scale bars are 50 μm.

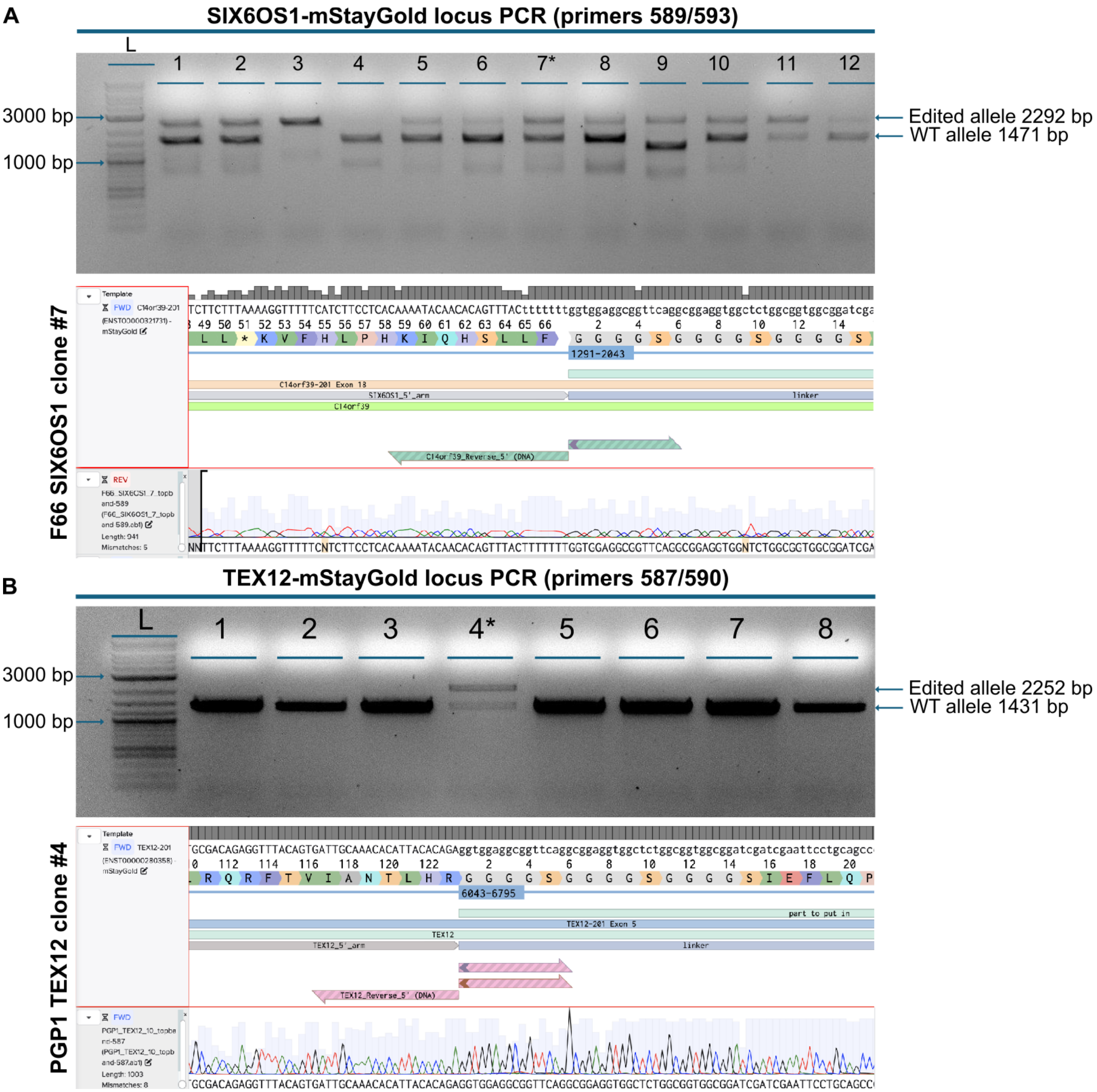

Figure S14. Genotyping PCR results for mStayGold reporters. Lines marked with an asterisk were used for experiments shown in Figure 6. PCR was performed in order to amplify the entire locus (both reporter and wild-type alleles). A view of the Sanger sequencing results at the 5' junction of the knock-in is shown for each line. (A) SIX6OS1-mStayGold knockin. (B) TEX12-mStayGold knockin.

id	Factor	Alternative names	Short description	Reason for inclusion	Barcodes
1	MEK1 S218D,S222D	MAP2K1	Constitutively active MEK1; promotes MAPK signaling	CPO library	TGACATTAAGCAATTGGTGA
2	myr-AKT1	PKB	Myristoylated AKT1; constitutively active form that promotes PI3K pathway signaling	CPO library	GACTGTCTCCTTGTTAAAAT
3	RHEB Q64L		Constitutively active RHEB; promotes mTOR signaling	CPO library	CTGTTTGGCTCCATTGGCCA
4	IKKa S176E,S180E	CHUK, IKK1	Constitutively active IKK alpha; promotes NF-κB signaling	CPO library	GAACGCGTTAAATTATTGCA
5	IKKb S177E,S181E	IKK2	Constitutively active IKK beta; promotes NF-κB signaling	CPO library	GAGTCCGCACCAGGGCCGGC
6	STAT3 A662C,N664C,V667L	APRF	Constitutively active STAT3; promotes JAK-STAT signaling	CPO library	TTCATATCGATCTTTAGCGC
7	CTNNB1 S33A,S37A,T41A,S45A	β-catenin	Degradation-resistant β-catenin; promotes WNT signaling	CPO library	AAATATTTATGACACCTGTA
8	GSK3β K85A		Dominant negative GSK3B; promotes WNT signaling	CPO library	TCCGCTGATCGCAGTTCCCT
9	CTNNB1 S33Y	β-catenin	Constitutively active CTNNB1; promotes WNT signaling	CPO library	GAGACATGCTGCTAAGCGGT
10	MAPK9	JNK2, SAPK	c-Jun N-terminal kinase 2; stress-activated protein kinase	CPO library	ATATCCGTCGCAGAACAGTT
11	MKK7-JNK2 fusion		Constitutively active JNK2 fusion protein	CPO library	TTAAAAACAATCAGTAAAGA
12	MEK5 DDS311D,T315D	MAP2K5	Constitutively active MEK5; promotes ERK5 signaling	CPO library	ATCGTAGCTGTCGTTGTTCG
13	MEK5	MAP2K5	ERK5 pathway kinase	CPO library	TGATGAGACTCTCATCATCA
14	Notch1 ICD	NICD1	Notch1 intracellular domain; constitutively active form	CPO library	GTAGTCACTTTTCCTTTTTG
15	Notch3 ICD	NICD3	Notch3 intracellular domain; constitutively active form	CPO library	TTATCATGTGCGTCCAAGAA
16	MAPK14	p38α	MAP kinase	CPO library	GAATTTGATCGTGGTATTA
17	MKK6 S207E,T211E	MAP2K6	Constitutively active MKK6; activates p38 MAPK	CPO library	TTCTCTAGAGTATTGTTGAA
18	GLI1 truncation		Constitutively active GLI1; promotes Hedgehog signaling	CPO library	CTGTAAATTGTGTTGTGCCG
19	SMOM2 W535L		Constitutively active Smoothened; promotes Hedgehog signaling	CPO library	GAAATATTTTCCTTGTTCCA
20	TGFβR1 T204D	ALK5	Constitutively active TGF-β receptor; promotes TGF-β signaling	CPO library	TCAAACGATCAAGTGAATGA
21	BCL2		Anti-apoptotic factor; regulates outer mitochondrial membrane permeability	CPO library	TTAATATCTGCTAAAAAAG
22	BCL-XL	BCL2L1	Anti-apoptotic factor; regulates outer mitochondrial membrane permeability	CPO library	TCGAGTGATTGTACGCGATA
23	Caspase-8 C360A	CASP8	Dominant negative caspase-8; inhibits extrinsic apoptosis pathway	CPO library	AAACAACAACGATCGAGTTA
24	Caspase-3 C163A	CASP3	Dominant negative caspase-3; inhibits apoptosis execution	CPO library	GTTATTCCAAAGTTACAAGT
25	ERα Y537S	ESR1	Constitutively active estrogen receptor	CPO library	GGGAGCTTTGGTAATCAAAC
26	AR-V7		Constitutively active androgen receptor	CPO library	TAGACCCTACGGTCAAACTA
27	YAP2-8SA		Constitutively active YAP2; promotes Hippo signaling	CPO library	GGCAAGGCTTACGACAGTGA
28	EZH2	KMT6	Histone methyltransferase; core component of PRC2 complex	Regulon analysis (oogonia_meiotic)	CCATGTGACAAATTCCCATC
29	KCTD19		Potassium channel tetramerization domain protein; role in spermatogenesis	Known from literature	AGAAGACCTTGATAAGCGCT

30	BRCA1	IRIS	Promotes DNA homologous recombination	Regulon analysis (oogonia_meiotic)	CAAGTTTGACAGACCTTCGA
31	DMRTB1	DMRT6	DM domain transcription factor; regulates meiosis	GRN analysis	TTGTAAGGCACACCGAGTCT
32	HMGB2	HMG2	High mobility group protein; chromatin remodeling factor	GRN analysis	GAACGGTCAACAACATTTAA
33	RAN		GTPase involved in nuclear transport	GRN analysis	GGCAGTGGTGAACGCAGATA
34	HMGB1	HMG1	High mobility group protein; chromatin and inflammation regulator	Regulon analysis (oogonia_STRA8)	CGAACAGTACACATCACGTC
35	HESX1	RPX	Homeobox transcription factor; developmental regulator	Regulon analysis (oogonia_meiotic)	TACCGAATGTAATGCCGCAC
36	RNF138	NARF	E3 ubiquitin ligase; active during meiosis	GRN analysis	TACAAAAAAATCATTTTTGA
37	HOXB5	HOX2A	HOX family transcription factor; role in meiosis not previously characterized	Regulon analysis (oogonia_STRA8)	GATGAGCGCTGCATTTTTAC
38	HOXA5	HOX1C	HOX family transcription factor; role in meiosis not previously characterized	Regulon analysis (oogonia_STRA8)	TTTTCTGGGGTGGCTCCCAA
39	HOXA10	HOX1H	HOX family transcription factor; role in meiosis not previously characterized	Regulon analysis (oogonia_STRA8)	TTTATCGTTTGTACAATGAA
40	HMGB3	HMG4	High mobility group protein; chromatin remodeling factor	Regulon analysis (oogonia_STRA8)	GCCGGTAACGTCGGTGACGA
41	DMC1	LIM15	Meiosis-specific recombinase; essential for meiotic recombination	GRN analysis	TGACGTTGGTTTGATAGCG
42	TERF1	PIN2	Telomeric repeat binding factor; regulates telomere length	GRN analysis	AGCAGGTTTATCTGATGCAG
43	ZCWPW1	ZCW1	Zinc finger protein; reads histone modifications in meiosis	Known from literature	TGACTAAGTATTCCATTGGC
44	RBM46		RNA binding protein; expressed in germ cells	Known from literature	AATGACTTTGAGCCAACAGA
45	RFX5		Regulatory factor X; transcription factor	Regulon analysis (oogonia_STRA8)	AAGAGGCACTTGCACTGTCA
46	CTCFL	BORIS	CTCF-like protein; male germline-specific transcription factor	Known from literature	TCTTTGTCGTATGGTGTCAA
47	MYBL1	A-MYB	Transcription factor essential for male meiosis	Regulon analysis (oogonia_meiotic)	TTTGTATATTCCGGCAATGT
48	DMRTC2	DMRT7	DM domain protein; regulates sexual development	GRN analysis	TTATGACTTTGACTTGACAA
49	ANHX		Anomalous homeobox protein; expressed in oogonia	GRN analysis	GGCAACGCACTTGACGCCAG
50	RB1	pRb	Retinoblastoma protein; cell cycle regulator	Regulon analysis (oogonia_meiotic)	ACCTTACTTAAAATCGTTTC
51	MEIOSIN		Meiosis initiator protein	Known from literature	CACACTCATTGAAACTTGA
52	SAP30		Sin3A-associated protein; transcriptional repressor	Regulon analysis (oogonia_meiotic)	TAAATGTGTGGGTAAAAGTT
53	HDAC2		Histone deacetylase 2; epigenetic regulator	Regulon analysis (oogonia_meiotic)	ATGCAATGCTAAGTTGGAGA
54	SMC3	CSPG6	Structural maintenance of chromosomes protein; cohesin complex	GRN analysis	TGCTTGTGAGTTTGGTGGAT
55	KDM5B	JARID1B	Histone demethylase; epigenetic regulator	Regulon analysis (oogonia_meiotic)	ACTGCGCTGTCTTTTTCAAA
56	SYMPK		Symplekin; involved in polyadenylation	Known from literature	GGTGCAGAAGCTAACTTTCC
57	MSX1	HOX7	Homeobox transcription factor; expressed during meiosis	Regulon analysis (oogonia_STRA8)	CATATTTAGTTCTCCTTCTT
58	YTHDC2		RNA m6A reader and helicase; expressed during meiosis	Known from literature	TGCCTATTATAGGGGTGCCG
59	NELFE	RDBP	Negative elongation factor E; transcription regulator	Regulon analysis (oogonia_STRA8)	TGAGCAATATACAAAAGACA
60	ZNF541	SHIP1	Zinc finger transcription factor; expressed during meiosis	Known from literature	CTTCATAAACCCATCGTTGA
61	NFYB	CBF-A	Nuclear transcription factor Y subunit beta	Regulon analysis (oogonia_meiotic)	GACTGTAACTGCGATTGCAA

62	MEIOC		Meiosis specific with coiled-coil domain	Known from literature	GATTTTGAGTACGATTACTC
63	ESX1	ESXR1	Homeobox protein; expressed in oogonia	GRN analysis	GAACCAGAAACAAAATAAAA
64	STRA8		Stimulated by retinoic acid 8; meiosis initiator	Known from literature	GGGCAGGGTAACGAGGGGGG
65	PRDM9		PR domain zinc finger protein 9; meiotic recombination hotspot determinant	GRN analysis	CCAGGAACTATCTATTCTTC
66	HDAC6		Histone deacetylase 6; cytoplasmic deacetylase	Regulon analysis (oogonia_meiotic)	GCCTTCCCTTCCAGATCCTA
67	RAD51	RECA	DNA homologous recombination protein; active during meiosis	GRN analysis	AAGACCATCTGACTCAAACA
68	SOD1		Superoxide dismutase 1; antioxidant enzyme	GRN analysis	ATTGGCGGTGCGCTCGCCGA
69	UBB		Ubiquitin B; protein degradation tag	GRN analysis	TGAATCACAGAAGAATACTC
70	RARG	NR1B3	Retinoic acid receptor gamma; assists in meiotic initiation	Regulon analysis (oogonia_STRA8)	TAAGGAAGTGAGCCAGATCT
71	NFYC	CBF-C	Nuclear transcription factor Y subunit gamma	Regulon analysis (oogonia_meiotic)	ATATTGCATGAAACCCTGTT; CACCGTTTCCCTGACTAAAA; GCTGCAATCTTGTAAGACCA
72	NOBOX	OG2X	Oogenesis homeobox protein	GRN analysis	AAATTCAAAGAAGATAAAAG; AAGATGTTAGAATCAAGAAG; GCCAACTACAATAAATATTC
73	FIGLA	FIG alpha	Folliculogenesis specific basic helix-loop-helix	GRN analysis	ATTGACTATGACACCGATGA; TTATTGGCGAGGTTAACAAC; GTAAATGTCCTTAATAACGT
74	DAZL	DAZH	Deleted in azoospermia-like; germ cell development	Known from literature	ACAGCGAACGAACATGATCT; GATATATTTAGTTGCTTCGC; AGTTTGTCTGTAAGCGAAAC
75	BOLL	BOULE	Boule-like protein; meiotic regulator	GRN analysis and known from literature	ATTGGGGGTAAGTCTATCTT; ATGATCATTGTATATACCGT; ATGGGGCTCGGAGCAGGACA
76	DDX4	VASA	DEAD-box helicase; germline development	GRN analysis and known from literature	CATAAGCTTCCTTGTCCTAC; GGTGCAGGAGCTAACTTTCC; TAAAAACCGGGCTTTTTCAC
77	ELK1		ETS domain-containing protein	Regulon analysis (oogonia_STRA8)	GCAGGAAGCGTTGTGTTGCA; ATCCCTTCTCACCAACCACC
78	TAF4B	TAFII105	TATA-box binding protein associated factor, germline-specific	Known from literature	ACTAACAATCCAGAACTTGC; TTCTTCAATGCTGGGTGATA
79	DNMT1 CRISPRi		Inhibits maintenance DNA methyltransferase	DNA demethylation	ATAGAGAGACCATTAATGGG TCTCCTAGG
80	DPPA3	STELLA	Developmental pluripotency associated protein 3; inhibits maintenance DNA methyltransferase	DNA demethylation	GATAGCATGACTTATGAAAT
81	TET1		TET family dioxygenase; actively demethylates DNA	DNA demethylation	GGTATTTTGCCCGTGATTCT
82	TET3		TET family dioxygenase; actively demethylates DNA	DNA demethylation	AGATCTCTAGAAATCTAAAA
83	SMAD1-active		Constitutively active SMAD1; BMP signaling effector	BMP signaling	AATACCTATCACTATAGCTT
84	SMAD9-active	SMAD8	Constitutively active SMAD9; BMP signaling effector	BMP signaling	CACAGAGGTCGGACGAAGTT
85	DMRT1		DM domain transcription factor; expressed in germ cells and male gonadal somatic cells	Known from literature	TTACTTCGGAAACTGAAGAG; TTATTGGAGACAACAACGCA
86	PAX5	BSAP	Paired box transcription factor	Known from literature	GCTGCCTCTCAGATTTCTGA
87	PAX6 isoform a	AN2	Paired box transcription factor	GRN analysis	CTTTTCAAATAAGACGTCTA
88	PAX6 isoform b		Alternative splice variant of PAX6	GRN analysis	ATTGAAGGACTTTTCAGTTA

Table S1. Factors screened for meiosis induction. #1-78 were included in barcode enrichment screening, and #79-88 were added for scRNAseq screening.

DMC1
HORMAD1
HORMAD2
INCA1
MEIOB
PRDM9
RAD51AP2
SCML1
SHCBP1L
SMC1B
SPATA22
SPDYA
SPO11
SYCE2
SYCE3
SYCP1
SYCP2
SYCP3
TEX12

Table S2. Genes used for meiosis gene score calculation.

Name	Sequence	Purpose
MPS542_Barcode pulldown_3'_F	/5BiosG/GTTTTAGAGCTAGAAATAGC	Barcode capture (scRNAseq)
MPS543_Barcode pulldown_3'_R	/5BiosG/TCCAAACTCATCAATGTATC	Barcode capture (scRNAseq)
MPS546_Illumina_p5	AATGATACGGCGACCACCGAGATCT	Barcode capture (scRNAseq)
MPS547_Illumina_p7	CAAGCAGAAGACGGCATACGAGAT	Barcode capture (scRNAseq)
oAMP123_barcode_F	ACACTCTTTCCCTACACGACGCTCTTCCGATCTNNATACTCAGAAGATGTCACTCACC	Barcode enrichment
oAMP124_barcode_R	GAGTTCAGACGTGTGCTCTTCCGATCTNNNCATTCATAGTTCTTGCTCAGTGG	Barcode enrichment
MPS017_REC8_5'arm_fwd	TGGAGCCCAGTGTTCCCCTTATG	Cloning
MPS018_REC8_seq_fwd	CAAGAAAAGCCATATGGTCGC	Cloning
MPS019_REC8_5'_rev	GTGGAATCTGGGCCCCGGCT	Cloning
MPS020_REC8_3'fwd	GGTTAGAGTCCATTTACAAAGCTGC	Cloning
MPS021_REC8_rev	TAGAAGTGGCCGGTTTCCTG	Cloning
MPS022_REC8_3'rev	TGCTCTCTGAAGAGATTTTGCC	Cloning
MPS458_BB_SYCP3 Gibson	caaataggggttccgcgcacatttccccgTCCGAGACCTAAATTGCGGG	Cloning
MPS459_SYCP3_mGL Gibson	GTTTTTTAAAGTCAGGAAGCATCTAAATATGGTGTCCaagggcgaggagctgttcac	Cloning
MPS460_SYCP3_5'_rev	GGACACCATATTTAGATGCT	Cloning
MPS461_T2A-SYCP3-Gibson	CCTGGAATACTTTTTTCCGGAGGACACCATtgggccaggattctcctcga	Cloning
MPS462_SYCP3_3'_fwd	ATGGTGTCCTCCGGAAAAAAG	Cloning
MPS463_SYCP3_BB Gibson	GAAAACCACACTGACAAGATCTGGAGGTTCTTCTATGTGAGAACAAGGCAT	Cloning
MPS568_mStayGold_TEX12_5_R	CACTGACAAGATCTGGAGGTACCACCTCTATCAACCCAAT	Cloning
MPS569_mStayGold_TEX12_5_F	ATTGGGTTGATAGAGGTGGTACCTCCAGATCTTGTCAGTG	Cloning
MPS570_mStayGold_TEX12_4_F	ttaGGCGCGCCGATATCTAGAGAATGACATTTATGCTTTGA	Cloning
MPS571_mStayGold_TEX12_4_R	CAAAGCATAAATGTCATTCTCTAGATATCGGCGCGCCt	Cloning
MPS572_mStayGold_TEX12_2_R	ccgcctgaaccgcctccaccTCTGTGTAATGTGTTTGCAATCAC	Cloning
MPS573_mStayGold_TEX12_2_F	TTGCAAACACATTACACAGAggtggaggcggttcagg	Cloning
MPS574_mStayGold_TEX12_1_F	gttccgcgcacatttccccgATTCTGTGGCTGCCAAACCT	Cloning
MPS575_mStayGold_TEX12_1_R	AGGTTTGGCAGCCACAGAATcggggaaatgtgcgc	Cloning
MPS576_mStayGold_SIX6OS1_1_R	CCAGAATTGCCATTAAGGAAcggggaaatgtgcgc	Cloning
MPS577_mStayGold_SIX6OS1_5_F	TCAAACATTCAACAACTGCTACCTCCAGATCTTGTCAGTG	Cloning
MPS578_mStayGold_SIX6OS1_5_R	CACTGACAAGATCTGGAGGTAGCAGTTGTTGAATGTTTGATG	Cloning
MPS579_mStayGold_SIX6OS1_4_R	GTAAATTTATGCCCTCATGACTAGATATCGGCGCGCCt	Cloning
MPS580_mStayGold_SIX6OS1_4_F	ttaGGCGCGCCGATATCTAGTCATGAGGGCATAAATTTACATTATT	Cloning
MPS581_mStayGold_SIX6OS1_3_F	tgccaattatttaaagttacatatgAGAAAGTATAGGAACTTCAGCTTG	Cloning
MPS582_mStayGold_SIX6OS1_3_R	actttaaataattggcattatttaaagttattacaggtgggcctccag	Cloning
MPS583_mStayGold_SIX6OS1_2_F	CAACACAGTTTACtttttttggtggaggcggttcagg	Cloning
MPS584_mStayGold_SIX6OS1_2_R	ccgcctgaaccgcctccaccaaaaaaaGTAAACTGTGTTGTATTTTGTG	Cloning
MPS585_mStayGold_SIX6OS1_1_F	gttccgcgcacatttccccgTTCCTTAATGGCAATTCTGGGC	Cloning
MPS166_REC8_1F_CRISPRa	caccgCCTGGCAACAGGGTCTCCCG	CRISPRa sgRNA
MPS167_REC8_1R_CRISPRa	aaacCGGGAGACCCTGTTGCCAGGc	CRISPRa sgRNA
MPS168_REC8_2F_CRISPRa	caccgTGACAGCCAATGGGGAACGG	CRISPRa sgRNA
MPS169_REC8_2R_CRISPRa	aaacCCGTTCCCCATTGGCTGTCAc	CRISPRa sgRNA
MPS170_REC8_3F_CRISPRa	caccGTCGGGAACGCCAAGTATCC	CRISPRa sgRNA
MPS171_REC8_3R_CRISPRa	aaacGGATACTTGGCGTTCCCGAC	CRISPRa sgRNA
MPS172_REC8_4F_CRISPRa	caccgCCTCGGGAGACCCTGTTGCC	CRISPRa sgRNA
MPS173_REC8_4R_CRISPRa	aaacGGCAACAGGGTCTCCCGAGGc	CRISPRa sgRNA
MPS479_SYCP3_F_CRISPRa	accGCGCCCAAATAGCTGGCCCA	CRISPRa sgRNA
MPS480_SYCP3_R_CRISPRa	aacTGGGCCAGCTATTTGGGCGC	CRISPRa sgRNA
MPS023_REC8_outer_5'	ACTTCTCCCATCCCCAGGTC	Genotyping
MPS024_REC8_outer_3'	GCCACCACACCATACATTTCAATC	Genotyping
MPS489_SYCP3_outer_5'	TGTCGAAATCTTCTGCCTGTGT	Genotyping
MPS490_SYCP3_outer_3'	ACCATTCACTCTTAACATCATGGA	Genotyping

MPS586_TEX12 _3'_outer	AATTACTTTGTTGACCTCCAACCT	Genotyping
MPS587_TEX12 _5'_outer	CTTGAACAGATGTGAGCAAGGA	Genotyping
MPS588_SIX6OS1_5'_inner	GGACAGGGTCAAAATTCAATACC	Genotyping
MPS589_SIX6OS1 _3'_inner	GCACACACAAAACCCAAaatattca	Genotyping
MPS590_TEX12_3'_inner	GGAGTTTCCCCTTCTCTATTCG	Genotyping
MPS591_TEX12 _5'_inner	GAACTCTTCAAAGAAGCCAATGCT	Genotyping
MPS592_SIX6OS1 _3'_outer	ACCAATCAATTCAGTCATAGAGTCA	Genotyping
MPS593_SIX6OS1 _5'_outer	atgaaaCATTCAAAGTGATGCAAAT	Genotyping
MPS594_mStayGold_3'_seq	ggagagagaccacatcatcc	Genotyping
MPS595_mStayGold_5'_seq	tcctcgcctgtagacaccat	Genotyping
MPS064_REC8_cterm sgRNA_1 FWD	caccGCCGGGGCCCAGATTCCACTG	Knock-in sgRNA
MPS065_REC8_cterm sgRNA_1 REV	aaacCAGTGGAATCTGGGCCCCGGC	Knock-in sgRNA
MPS066_REC8_cterm sgRNA_2 FWD	caccGTAAATGGACTCTAACCTCAG	Knock-in sgRNA
MPS067_REC8_cterm sgRNA_2 REV	aaacCTGAGGTTAGAGTCCATTTAC	Knock-in sgRNA
MPS068_REC8_cterm sgRNA_4 REV	aaacGGGCCCAGATTCCACTGAGGC	Knock-in sgRNA
MPS069_REC8_cterm sgRNA_4 FWD	caccGCCTCAGTGGAATCTGGGCCC	Knock-in sgRNA
MPS070_REC8_cterm sgRNA_5 REV	aaacGATTCCACTGAGGTTAGAGTC	Knock-in sgRNA
MPS071_REC8_cterm sgRNA_5 FWD	caccGACTCTAACCTCAGTGGAATC	Knock-in sgRNA
MPS072_REC8_cterm sgRNA_3 REV	aaacAGATTCCACTGAGGTTAGAGC	Knock-in sgRNA
MPS073_REC8_cterm sgRNA_3 FWD	caccGCTCTAACCTCAGTGGAATCT	Knock-in sgRNA
MPS464_SYCP3_sgRNA_fwd	caccGCATCTAAATATGGTGTCCTC	Knock-in sgRNA
MPS465_SYCP3_sgRNA_rev	aaacGAGGACACCATATTTAGATGC	Knock-in sgRNA
MPS560_sgRNA_TEX12	aaacATTATTGTTATAATGAAAGAC	sgRNA
MPS561_sgRNA_TEX12	CACCGTCTTTCATTATAACAATAAT	sgRNA
MPS562_sgRNA_SIX6OS1_1	aaacTCATGAACACAGAACAGTAAC	sgRNA
MPS563_sgRNA_SIX6OS1_1	caccGTTACTGTTCTGTGTTCATGA	sgRNA
MPS564_sgRNA_SIX6OS1_2	aaacCATGAACACAGAACAGTAAAC	sgRNA
MPS565_sgRNA_SIX6OS1_2	caccGTTTACTGTTCTGTGTTCATG	sgRNA
MPS566_sgRNA_SIX6OS1_3	aaacTAAATTATTTTACTGTTCTGC	sgRNA
MPS567_sgRNA_SIX6OS1_3	caccGCAGAACAGTAAAATAATTTA	sgRNA
qMPS001F_GAPDH	GGTGACCAGGCGCCCAATACGA	qPCR
qMPS001R_GAPDH	CGCTTCGCTCTCTGCTCCTCCTGT	qPCR
qMPS070F_DPPA3	ACGCCGATGGACCCATCACAGTTT	qPCR
qMPS070R_DPPA3	TCTCGGAGGAGATTTGAGAGGCCC	qPCR
qMPS071F_REC8	TACCTGCTCCTGGTGCTCTC	qPCR
qMPS071R_REC8	TGGATCAGGAGGCGACCATA	qPCR
qMPS072F_SYCP3	TCTACTTACTGGTGCACAAAATGA	qPCR
qMPS072R_SYCP3	TCTCTTGCTGCTGAGTTTCCA	qPCR
qMPS073F_MAX	GGACTCGGCTTGTTGTTGTC	qPCR
qMPS073R_MAX	GGTTGCTCTTCGTCGCTCT	qPCR
qMPS074F_MGA	ACCGAACAGAATAACCCGCC	qPCR
qMPS074R_MGA	AATGGCCTTCCCATCCGTG	qPCR
qMPS075F_E2F6	ACCCAGTCTCCTCCTGGAC	qPCR
qMPS075R_E2F6	TATTTTTGATGGCAGCAGGC	qPCR
qMPS076F_RNF2	GCAGGAGCCGCAATGTCT	qPCR
qMPS076R_RNF2	ATTGCCTCCTGAGGTGTTCG	qPCR
qMPS077F_PCGF6	GAGGACGAGGACGAGGAGTT	qPCR
qMPS077R_PCGF6	GATTAATCAGGCGCTCCTCC	qPCR
qMPS078F_DNMT1	CCCCAAAGAACCAACGGAGA	qPCR
qMPS078R_DNMT1	CTGAATGCACTTGGGAGGGT	qPCR
qMPS079F_DNMT3A	CACCGGCCATACGGTGGAG	qPCR
qMPS079R_DNMT3A	TGTTGAGCCCTCTGGTGAAC	qPCR
qMPS080F_DNMT3B	GGAGATTCGCGAGCCCAG	qPCR
qMPS080R_DNMT3B	CTCCCTTCATGCTTTCCTGC	qPCR
qMPS081F_UHRF1	ACAGGGGCAAACAGATGGAG	qPCR
qMPS081R_UHRF1	TGGATGGTGTCATTCAGGCG	qPCR
qMPS082F_SETDB1	GTTGTGAGTCTGGGGTCTGG	qPCR
qMPS082R_SETDB1	ATGCTTTTGTCCTCTCCCGT	qPCR

Table S3. Oligos used in this study.

Primary Antibodies					
**Target**	**Antibody type**	**Supplier**	**Cat. Number**	**Dilution**	**RRID**
SYCP3	Rabbit IgG, polyclonal	Abcam	ab15093	1:250	AB_301639
DDX4	Rabbit IgG, polyclonal	Abcam	ab13840	1:250	AB_443012
KI67	Rat IgG, monoclonal	Thermo	14-5698-37	1:250	AB_2865119
HORMAD1	Rabbit IgG, polyclonal	Proteintech	13917-1-AP	1:250	AB_2120844
γH2AX	Mouse IgG, monoclonal	Millipore Sigma	05-636	1:500	AB_309864
Centromere	Human IgG, polyclonal, FITC-labeled	Antibodies Inc	15-235-F	1:200	AB_2797147
SYCP3	Goat IgG, polyclonal	R&D Systems	AF3750	1:250	AB_2197194
T2A	Rat IgG, monoclonal	Millipore Sigma	MABE1923	1:250	AB_3097817
TEX12	Rabbit IgG, polyclonal	Abcam	ab122455	1:250	AB_11128111
RAD51	Mouse IgG, monoclonal	Novus	NB100-148	1:250	AB_10002131
SUN1	Rabbit IgG, polyclonal	Novus	NBP1-87396	1:250	AB_11025926
MSH4	Rabbit IgG, polyclonal	Thermo	PA5-115593	1:250	AB_2900228
MLH1	Mouse IgG, monoclonal	BD	554073	1:100	AB_395227
TEX14	Mouse IgG, monoclonal, CF488-labeled	Proteintech	CL488-67982	1:100	AB_2923849
SYCE1	Rabbit IgG, polyclonal	Proteintech	17406-1-AP	1:250	AB_10597113
**Secondary antibodies (all donkey polyclonal)**					
**Target**	**Fluorophore**	**Supplier**	**Cat. Number**	**Dilution**	**RRID**
Goat IgG	AF568	Thermo Fisher	A11057	1:500	AB_2534104
Mouse IgG	AF488	Jackson	715-546-151	1:500	AB_2340850
Mouse IgG	AF647	Thermo Fisher	A31571	1:500	AB_162542
Rabbit IgG	AF488	Jackson	711-545-152	1:500	AB_2313584
Rabbit IgG	AF568	Thermo Fisher	A10042	1:500	AB_2534017
Rabbit IgG	AF647	Jackson	711-607-003	1:500	AB_2340626
Rat IgG	Dylight755	Thermo Fisher	SA5-10031	3:500	AB_2556611

Table S4. Antibodies used in this study.

#	Cell line	Factors	Sorting	Total Cells	Germ cell	Germ cell mitotic	PGC	Oogonia STRA8	Oogonia meiotic	Pre-oocyte	Pre-spermat ogonia
1	PGP1 D4TR8G	BCL2, HOXB5, and STRA8	unsorted	3553	1323	350	257	1605	0	0	18
2	F3 D4TS3G	BCL2, HOXB5, and STRA8	unsorted	5308	1187	320	299	3464	4	0	34
3	PGP1 D4TR8G	BCL2, HOXB5, STRA8, and the pool of 88 factors	unsorted	90809	32279	10728	9246	37839	27	1	689
4	PGP1 D4TR8G	BCL2, HOXB5, STRA8, and the pool of 88 factors	sorted DDX4+	59668	20629	6630	6399	24732	65	6	1207
5	PGP1 D4TR8G	BCL2, HOXB5, STRA8, and the pool of 88 factors	sorted REC8+	76522	26398	11405	9701	28595	14	0	409
6	F3 D4TS3G	BCL2, HOXB5, STRA8, and the pool of 88 factors	unsorted	50837	14834	4481	3267	27916	38	1	300
7	F3 D4TS3G	BCL2, HOXB5, STRA8, and the pool of 88 factors	sorted DDX4+	59742	14949	6030	5233	32593	100	4	833
8	F3 D4TS3G	BCL2, HOXB5, STRA8, and the pool of 88 factors	sorted SYCP3+	88342	23339	6991	5549	51541	238	6	678
9	PGP1 D4TR8G	Pool of 88 factors	unsorted	10069	3607	2197	2213	2019	1	0	32
10	PGP1 D4TR8G	Pool of 88 factors	sorted DDX4+	28038	9798	6119	5401	6617	2	0	101
11	PGP1 D4TR8G	Pool of 88 factors	sorted REC8+	27982	10776	5316	5497	6114	6	1	272
12	F3 D4TS3G	Pool of 88 factors	unsorted	35747	9359	9648	9883	6772	1	0	84
13	F3 D4TS3G	Pool of 88 factors	sorted DDX4+	25380	10364	4287	4396	5996	11	1	325
14	F3 D4TS3G	Pool of 88 factors	sorted SYCP3+	32779	12425	5816	5202	8966	34	0	336
15	Mix of PGP1 D4TR8G and F3 D4TS3G	No factors	unsorted	5312	1227	1880	2016	189	0	0	0

Table S5. List of samples in the scRNAseq screening experiment, and numbers of annotated cell types.

Treatment	Treatment Explanation	SYCP3+ (mean)	SYCP3+ (stdev)	HORMAD1 + (mean)	HORMAD1 + (stdev)	γH2AX+ (mean)	γH2AX+ (stdev)
All Negative	No media supplements (just APEL2 basal medium)	0.00%	0.00%	0.00%	0.00%	2.16%	1.89%
All Positive	All media supplements (5 μM GSK3484862 (DNMT1i), 1 μM AM580, 1 μg/mL doxycycline, 500 nM Shield1)	15.16%	11.82%	8.18%	1.99%	9.61%	4.13%
DNMTi Negative	Leaving out DNMTi completely. All other supplements at full dose.	0.00%	0.00%	0.00%	0.00%	0.00%	0.00%
DNMTi Remove Day 5	Leaving out DNMTi starting from day 5. All other supplements at full dose.	21.38%	27.76%	13.97%	24.09%	4.90%	5.46%
DNMTi Remove Day 7	Leaving out DNMTi starting from day 7. All other supplements at full dose.	11.43%	11.73%	8.83%	6.64%	6.85%	2.31%
DNMTi Remove Day 9	Leaving out DNMTi starting from day 9. All other supplements at full dose.	11.51%	11.59%	10.61%	7.09%	10.10%	3.97%
AM580 Day 1	Leaving out AM580 until day 1, then adding it afterwards. All other supplements at full dose.	16.70%	15.78%	9.87%	13.03%	9.49%	5.31%
AM580 Day 3	Leaving out AM580 until day 3, then adding it afterwards. All other supplements at full dose.	15.51%	8.79%	9.33%	4.55%	4.63%	2.01%
AM580 Day 5	Leaving out AM580 until day 5, then adding it afterwards. All other supplements at full dose.	5.71%	3.51%	6.95%	5.51%	3.65%	2.47%
AM580 Day 7	Leaving out AM580 until day 7, then adding it afterwards. All other supplements at full dose.	4.04%	4.53%	1.80%	0.77%	2.38%	2.07%
AM580 Day 9	Leaving out AM580 until day 9, then adding it afterwards. All other supplements at full dose.	10.87%	12.34%	8.06%	8.40%	5.03%	5.44%
AM580 Negative	Leaving out AM580 completely. All other supplements at full dose.	2.84%	3.44%	1.91%	0.28%	2.10%	2.23%
SHIELD1 Day 1	Leaving out Shield1 until day 1, then adding it afterwards. All other supplements at full dose.	11.20%	13.87%	12.99%	10.11%	4.99%	2.12%
SHIELD1 Day 3	Leaving out Shield1 until day 3, then adding it afterwards. All other supplements at full dose.	10.59%	9.44%	7.28%	7.44%	8.63%	6.91%
SHIELD1 Day 5	Leaving out Shield1 until day 5, then adding it afterwards. All other supplements at full dose.	11.99%	13.61%	5.00%	6.47%	7.09%	1.40%
SHIELD1 Day 7	Leaving out Shield1 until day 7, then adding it afterwards. All other supplements at full dose.	14.42%	17.27%	9.16%	12.52%	2.84%	2.33%
SHIELD1 Day 9	Leaving out Shield1 until day 9, then adding it afterwards. All other supplements at full dose.	16.81%	11.36%	6.54%	4.07%	5.29%	3.05%
SHIELD1 Negative	Leaving out Shield1 completely. All other supplements at full dose.	10.98%	10.15%	5.97%	1.94%	7.46%	1.11%
Dox Day 1	Leaving out doxycycline until day 1, then adding it afterwards. All other supplements at full dose.	19.58%	7.51%	29.08%	7.19%	7.96%	8.88%
Dox Day 3	Leaving out doxycycline until day 3, then adding it afterwards. All other supplements at full dose.	6.93%	8.38%	10.19%	11.09%	10.22%	8.05%
Dox Day 5	Leaving out doxycycline until day 5, then adding it afterwards. All other supplements at full dose.	13.29%	12.10%	1.88%	1.46%	3.49%	1.87%
Dox Day 7	Leaving out doxycycline until day 7, then adding it afterwards. All other supplements at full dose.	5.61%	4.40%	0.77%	0.28%	2.58%	2.66%
Dox Day 9	Leaving out doxycycline until day 9, then adding it afterwards. All other supplements at full dose.	3.08%	2.51%	1.61%	2.28%	3.02%	0.60%
Dox Negative	Leaving out doxycycline completely. All other supplements at full dose.	10.97%	13.73%	23.65%	39.35%	4.16%	2.28%
Dox Zero SHIELD Low	No doxycycline, 100 nM Shield1. All other supplements at full dose.	3.81%	3.76%	0.15%	0.30%	2.91%	2.17%
Dox Low SHIELD Zero	0.1 μg/mL doxycycline, no Shield1. All other supplements at full dose.	12.01%	5.95%	4.34%	1.02%	6.04%	1.45%
Dox Low SHIELD Low	0.1 μg/mL doxycycline, 100 nM Shield1. All other supplements at full dose.	17.83%	16.37%	7.02%	4.94%	8.06%	5.16%
Dox Low SHIELD High	0.1 μg/mL doxycycline, 500 nM Shield1. All other supplements at full dose.	15.77%	18.58%	9.63%	11.36%	7.77%	3.05%
Dox High SHIELD Low	1 μg/mL doxycycline, 100 nM Shield1. All other supplements at full dose.	18.35%	17.71%	20.15%	28.42%	7.82%	2.47%
Sequential 1	Shield1 added only from days 0–5, doxycycline added only from days 5–11. All other supplements at full dose.	11.36%	11.89%	7.42%	6.39%	6.49%	2.39%
Sequential 2	Shield1 added only from days 0–7, doxycycline added only from days 5–11. All other supplements at full dose.	15.12%	12.36%	2.38%	1.83%	6.51%	5.46%
Sequential 3	Shield1 added only from days 0–7, doxycycline added only from days 7–11. All other supplements at full dose.	8.10%	7.51%	0.33%	0.57%	2.68%	1.07%

Table S6. List of media supplement dosages and timings tested for meiosis induction over an 11-day protocol, with doxycycline-inducible BOLL and Shield1-inducible HOXB5 (related to Fig. S6). Mean percentages and standard deviations of marker-positive cells are listed for SYCP3, HORMAD1, and γH2AX.

Feature class	Fold enrichment	Total peak bases	Total feature bases	Total bases overlapping	% feature coverage
Promoters	5.5	274980500	38477400	20089852	52.2
CpG islands	6.0	274980500	24827977	14153415	57.0
ICRs	4.6	274980500	586877	254643	43.4
TEs	0.6	274980500	1634149049	94177842	5.8

Table S7. Coverage and fold enrichment of ATAC peaks for different classes of genomic features (promoters, CpG islands, imprint control regions, and transposable elements).

Table S8. (separate file)

List of differentially accessible regions, enriched gene ontology terms, TF motifs, and regulons from single cell multi-omics data.

Table S9. (separate file)
Sequences of plasmids used in this study.

Movie S1. (separate file)
3D projection of Z-stack imaging of day 15 male meiotic cells (depicted in Figure 5A).
